# CD73 polymorphisms are associated with schizophrenia

**DOI:** 10.1007/s11302-024-10004-3

**Published:** 2024-05-17

**Authors:** He-Xia Peng, Li-Li Zhang, Dan Jiang, Na Jian, Ting-Mei Zhang, Jia-Guo Luo, Hai-Yan Yin

**Affiliations:** 1 School of Acupuncture and Tuina, Chengdu University of Traditional Medicine, Chengdu, 610075 China; 2 Chengdu Jinxin Mental Diseases Hospital, Chengdu, 610063 China

**Keywords:** CD73, Schizophrenia, Single-nucleotide polymorphism, Ecto-5’-nucleotidase

## Abstract

Ecto-5’-nucleotidase/CD73 enzyme plays a key role in the regulation of extracellular adenosine levels, thereby exerting influence on adenosine homeostasis. Emerging evidence suggests that perturbations in purines and ecto-5’-nucleotidase activity are associated with an augmented susceptibility to schizophrenia. However, the precise impact of genetic variations in CD73 on individuals with schizophrenia remains poorly understood. Here, our study demonstrated that rs3734442 allele and rs4431401 heterozygote were conferred a significant risk of schizophrenia disease (rs3734442: odds ratio, 0.556; 95% CI, 0.375 to 0.825; *p* = 0.004; rs4431401: odds ratio, 1.881, 95% CI, 1.117 to 3.166; *p* = 0.020). Comparing different genders, we observed a significant association between rs3734442 genotypes and male cases (rs3734442: odds ratio, 0.452; 95% CI, 0.257 to 0.796; *p* = 0.007). Likewise, there was a significant association between rs4431401 genotypes and male patients (rs4431401: odds ratio, 2.570; 95% CI, 1.196 to 5.522; *p* = 0.015). Based on family history and antipsychotics medication usage, our data reveals that the rs9444348 allele exhibits the most significant association with familial susceptibility to schizophrenia (odds ratio, 1.541; 95% CI, 1.009 to 2.353; *p* = 0.048 for A vs G). Moreover, individuals carrying variants of rs6922, rs2229523, and rs2065114 while being treated with clozapine demonstrate a higher frequency proportion compared to those receiving risperidone treatment (*p* = 0.035; *p* = 0.049; *p* = 0.027 respectively). Additionally, our results indicate that patients with GG genotype of rs9444348 had significantly higher likelihood of using clozapine instead of sulpiride (*p* = 0.048). Overall, our data strongly suggest that genetic variations in CD73 are significantly associated with schizophrenia risk and may serve as valuable resources for identifying therapeutic targets.

**Supplementary Information:**

The online version contains supplementary material available at 10.1007/s11302-024-10004-3.

## Introduction

Schizophrenia (SCZ) is a debilitating psychiatric disorder characterized by the presence of psychotic symptoms, including hallucinations and delusions, as well as impaired speech function. It also involves negative symptoms such as reduced motivation, flattened affect, social withdrawal, anhedonia, and cognitive deficits in executive functioning and working memory [1–3]. The onset of schizophrenia typically occurs during early adulthood and affects approximately 1% of the global population with a lifetime risk ranging from 0.3% to 0.66% [4, 5]. Long-term antipsychotic therapy is often necessary for managing schizophrenia; however, it carries an increased risk of adverse side effects for patients. Importantly, the challenges in the treatment and prevention of schizophrenia largely stem from the fact that its pathogenesis remains elusive [6].

Single nucleotide polymorphisms (SNPs), which are the most prevalent genetic variations in the human genome, have been found to play a significant role in the genetic mechanism underlying schizophrenia [7, 8]. Recent studies have suggested that different subtypes of purinergic receptors may contribute to the development of schizophrenia [9]. However, as a critical regulator in the purinergic signaling pathway, genetic deficiency of ecto-5’-nucleotidase (CD73) has been shown to alleviate neuronal degeneration and improve hippocampal-dependent memory manifestation [10]. Therefore, confirming the presence of genetic variations in CD73 gene may help explain the variability in schizophrenia risk and identify potential targets for future clinical intervention.

CD73/ecto-5’-nucleotidase plays a pivotal role in the synthesis of extracellular adenosine from AMP and serves as a major regulator of extracellular nucleotide hydrolysis [11, 12]. Extracellular adenosine, generated through CD73 activity, interacts with four G-protein coupled receptors (A1, A2A, A2B, and A3) to initiate distinct downstream signaling pathways and modulate adenylate cyclase activity [13–15]. The activation of A2AR has been considered as a potential strategy for developing atypical antipsychotic drugs targeting positive symptoms [16]. Recent research has suggested an association between schizophrenia risk and genetic variation at a single nucleotide polymorphism (SNP) within the adenosine A2R (ADORA2A) gene [17]. Furthermore, studies on patients with schizophrenia have indicated that SNPs in the A1 receptor gene are linked to the pathophysiological mechanisms underlying this disorder [18]. Previous investigations have demonstrated that inhibition of CD73 or its deficiency leads to significantly reduced levels of extracellular adenosine in rodents [19, 20]. Another study verified that mice with decreased extracellular adenosine levels exhibit locomotor and cognitive impairments resembling those observed in schizophrenia [21]. Therefore, differential regulation of extracellular adenosine levels by CD73 represents a potentially valuable set of biomarkers for assessing schizophrenia. However, there is limited understanding regarding the relationship between genetic variations associated with purinergic signaling components such as CD73 and schizophrenia.

Briefly, studies have confirmed the significance of ecto-5’-nucleotidase in the etiology of schizophrenia through rodent experiments; however, limited research has explored the association between genetic variation and schizophrenia. Our hypothesis posits that genetic variations in the CD73 gene may increase the risk of developing schizophrenia. Therefore, we investigated the distribution of genotypes and alleles in the CD73 gene between individuals with schizophrenia and healthy controls.

## Methods

### Study Population

242 cases with schizophrenia were recruited from October 2021 to October 2022. Among these participants, 143 (59.1%) were male, and 99 (40.9%) were female; The inclusion criterion for cases were identified with Diagnostic and Statistical Manual of Mental Disorders, fifth edition (DSM-5) [22]. The mean and standard error of the patients' age was 49.03 ± 0.711 years; The mean age at disease onset was 29.69 ± 0.583 years; The mean duration of illness was 19.33 ± 0.711 years; 60(24.8%) was family history of schizophrenia, and 182(75.2%) was without family history of schizophrenia; The utilization rates of antipsychotic medications were 21.9% for clozapine, 30.1% for risperidone, 9.1% for sulpiride, and 14.5% for other antipsychotics. Subjects were excluded if they had other major psychiatric disorders, pregnant or lactating woman, infectious diseases and were unable to provide signature. Controls were recruited from the staff in the same region according to the distribution of cases. The healthy control group consisted of 97 subjects, with 46 (47.4%) males and 51 (52.6%) females recruited based on the absence of a family history of schizophrenia or other mental illness, no exposure to contagion, and non-pregnant or non-lactating status. The mean age for the control group was 46.64 ± 1.052 years.

The study was approved by the Ethics Committee of Chengdu Jinxin Mental Diseases Hospital in Sichuan Province, China. Prior to participation, all subjects provided written consent for their inclusion in the study.

### SNP Selection

SNP were utilized for to assess the variability across the CD73 gene. A total of thirty SNPs were examined, including rs9444348, rs6922, rs4431401, rs2229523, rs4579322, rs9450282, rs3734442, rs2065114, rs4373337, rs4458647, rs9450279, rs142302523, rs144566178, rs245445215, rs185955009, rs189229964, rs190317062, rs191551083, rs192915959, rs200250022, rs369010777, rs370583260, rs373210294, rs375481678, rs3767922488, rs527285211, rs550934748, rs555116698, rs778453280, and rs1346768186. Among 30 SNPs, 5 SNPs (rs4431401, rs9444348, rs9450282, rs6922, and rs2229523) were selected from the publication (Shi NR, Wang Q, et al.; Diamond ML, Ritter AC, et al.; Stremitzer S, Sunakawa Y, et al.; Tokunaga R, Cao S, et al.). The remaining 25 SNPs were selected randomly from the NCBL database. The exclusion of rs245445215 was due to the unavailability of suitable detection sites, resulting in the analysis being limited to only 29 SNPs.

### DNA Extraction

The DNA was extracted from blood samples collected from each participant's cubital vein. The blood was stored in ethylenediaminetetraacetic acid (EDTA) tubes, and the DNA extraction was performed using the Qiagen kit (Qiagen, Hilden, Germany). The DNA samples were stored at -80℃ until further use.

### SNP Genotyping

All SNPs were genotyped following the iPLEX Gold Application Guide, which is widely recognized for its accuracy and reliability. The design of extension primers and PCR amplification for these SNPs was meticulously conducted using MassARRAY Assay Design v4.0 software, renowned for its precision in primer design. Furthermore, PCR reactions and extensions were performed strictly according to the manufacturer’s instructions to ensure consistency and reproducibility. Data analysis was carried out utilizing cutting-edge MALDI-TOF (matrix-assisted laser desorption/ionization-time of flight) technology with TYPER 4.0 software from Agena Bioscience, San Diego, CA, USA- a trusted platform in genetic research.

### Statistical Analysis

Statistical analyses were conducted using SPSS version 26.0 software (Chicago, IL, USA). Differences in demographic characteristics between patients with schizophrenia and control participants were assessed using the nonparametric independent samples Wilcoxon signed-rank test for continuous variables and the chi-square (χ^2^) test for categorical data. The deviation from Hardy–Weinberg equilibrium was examined through χ^2^ analysis. Fisher’s exact test or χ^2^ was employed to compare statistical differences in genotype and allele frequencies between subjects and controls. Significant variations were defined as two-tailed* p* < 0.05 in conditional analysis. Odds ratios (ORs) along with their corresponding 95% confidence interval (CIs) were calculated for each genotype and allele frequency.

## Results

### Hardy–Weinberg equilibrium

The Hardy–Weinberg equilibrium test was used to assess the genotype distribution difference between the schizophrenia patients and healthy controls with the observed and predicted values. According to the clustering performance and the description that A + B > 90%, 8 SNPs were selected for analysis, namely rs3734442, rs9444348, rs4431401, rs6922, rs2229523, rs4579322, rs9450282 and rs2065114.The samples of the two groups for these SNPs were the same, these loci did not deviate from the Hardy–Weinberg equilibrium, indicating that genetic balance had been achieved (P > 0.05). Hardy–Weinberg equilibrium analysis of the eight loci was shown in Supplementary Table S1.

### Demographic Characteristics

The patient demographic data was obtained from the electronic medical record. Demographic variable, including age, gender, disease progression, age of onset, family history, contagion status, and antipsychotic medication usage were recorded. A total of 279 patients with schizophrenia participated in the study; however, 37 cases of mental retardation were excluded from the analysis. Participant characteristics are summarized in Table 1.

**Table1:** Demographic clinical characteristics of the study population

Variables	Schizophrenia patients (*n* = 242)	Healthy controls (*n* = 97)	*P* value
Age (years)	49.03 ± 0.711	46.64 ± 1.052	0.068
Gender			
Male	143(59.1%)	46(47.4%)	0.054
Female	99(40.9%)	51(52.6%)	
Family history			
Yes	60(24.8%)	-	-
No	182(75.2%)	-	-
Age of onset	29.69 ± 0.583	-	-
Duration of illness (years)	19.33 ± 0.711	-	-
Monotherapy			
Clozapine	53(21.9%)	-	-
Risperidone	73(30.1%)	-	-
Sulpiride	22(9.1%)	-	-
Others antipsychotic drug	35(14.5%)	-	-
Polytherapy	59(24.4%)	-	-

* Plus-minus values are mean ± standard error of the mean; Data are presented as n (%)

* Chi-square analyses (c^2^) for categorical variables

### Linkage disequilibrium analysis

**Fig. 1:**
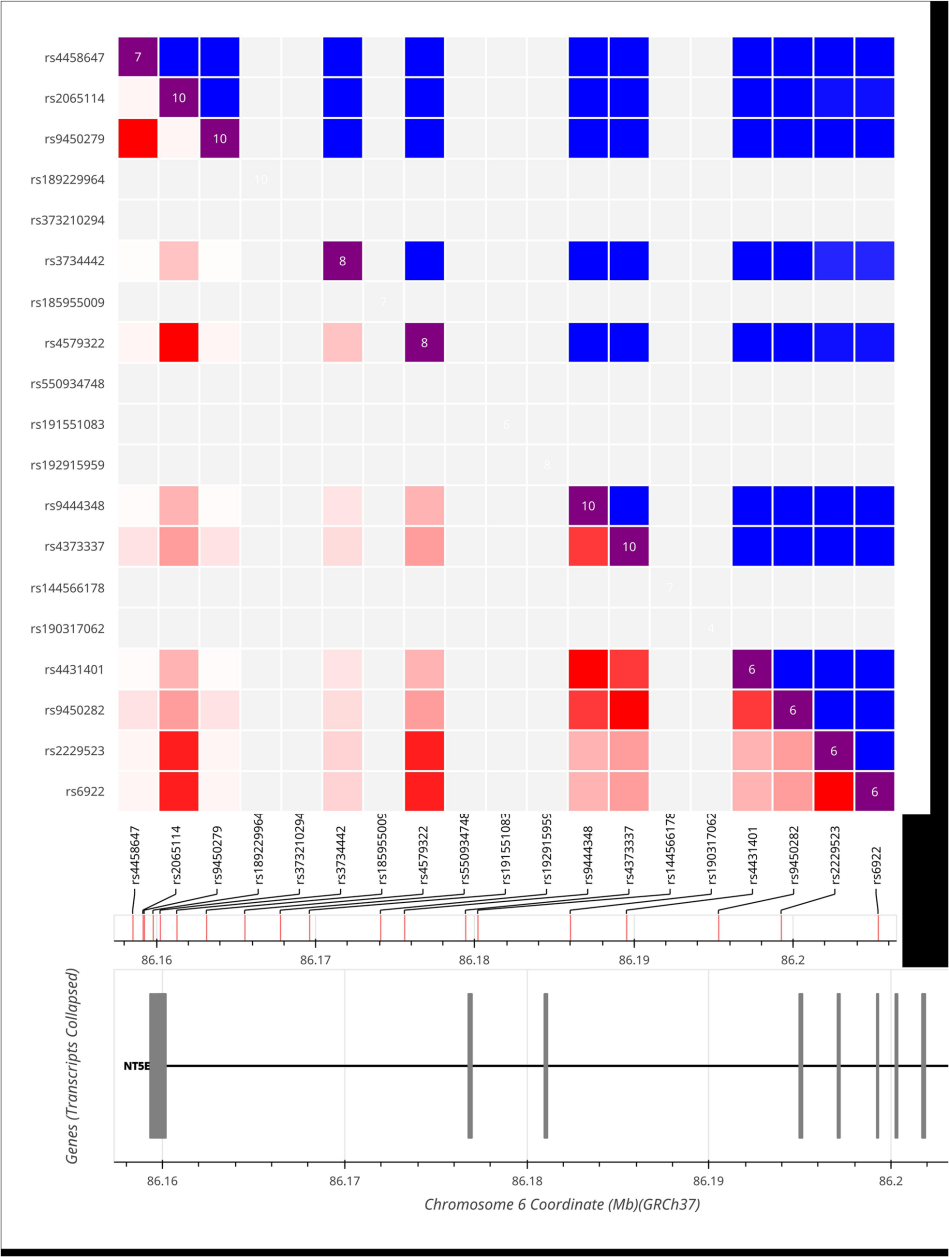
Linkage disequilibrium of CD73 SNPs loci

Non-random associations between various alleles within a group are known as linkage disequilibrium and are represented by D´ and r^2^. D´ has a range of 0 to 1. The degree of linkage disequilibrium increases with proximity to 1, with D´ equal to 0, the linkage equilibrium level. The linkage disequilibrium study of the eight loci indicates that there is clear linkage disequilibrium among these CD73 SNPs loci (Fig. 1).

### Evaluation of CD73 SNPs and schizophrenia risk

The frequencies of the genotypes and alleles of the CD73 polymorphisms in schizophrenia patients and control subjects are presented in Table 2 and Supplementary Table S2. Among the eight SNPs, individuals homozygous for the genotype at rs3734442 (AA) exhibited a significantly higher prevalence among patients with schizophrenia compared to healthy groups (OR = 0.207, 95% CI = 0.056–0.763, *p* = 0.019 for GG vs AA); Additionally, the TC genotype at rs4431401 was more prevalent among cases with schizophrenia than healthy groups (OR = 1.881, 95% CI = 1.117–3.166, *p* = 0.020 for TC vs TT) (Table 2). However, no significant associations were observed between the remaining evaluated SNPs and risk of schizophrenia as indicated in Supplementary Table S2.

**Table 2:** Genotypic and allelic distribution of the CD73 gene between schizophrenia patients and healthy controls

SNP	Genetic model	Genotype/allele	Schizophrenia patients	Healthy controls	OR	95% CI	P value
rs3734442	Codominant	GG vs AG vs AA	4(1.7%)/75(31.3%)/161(67.1%)	6(6.2%)/41(42.3%)/50(51.5%)	-	-	0.007*
	Allele	G vs A	83(17.3%)/397(82.7%)	53(27.3%)/141(72.7%)	0.556	0.375–0.825	0.004*
	Dominant	GG + AG vs AA	79(32.9%)/161(67.1%)	47(48.5%)/50(51.5%)	0.522	0.323–0.844	0.009*
	Recessive	GG vs AG + AA	4(1.7%)/236(98.3%)	6(6.2%)/91(93.8%)	0.257	0.071–0.932	0.037*
	Heterozygote	AG vs AA	75(31.8%)/161(68.2%)	41(45.1%)/50(54.9%)	0.568	0.346–0.933	0.029*
	Homozygote	GG vs AA	4(2.4%)/161(97.6%)	6(10.7%)/50(89.3%)	0.207	0.056–0.763	0.019*
	Additive	GG + AA vs AG	165(68.8%)/75(31.3%)	56(57.7%)/41(42.3%)	1.611	0.990–2.620	0.058
rs4431401	Codominant	CC vs TC vs TT	32(13.2%)/111(45.9%)/99(40.9%)	14(14.4%)/31(32.0%)/52(53.6%)	-	-	0.056
	Allele	C vs T	175(36.2%)/309(63.8%)	59(30.4%)/135(69.6%)	1.296	0.906–1.853	0.180
	Dominant	CC + TC vs TT	143(59.1%)/99(40.9%)	45(46.4%)/52(53.6%)	1.669	1.039–2.682	0.040*
	Recessive	CC vs TC + TT	32(13.2%)/210(86.8%)	14(14.4%)/83(85.6%)	0.903	0.459–1.779	0.861
	Heterozygote	TC vs TT	111(52.9%)/99(47.1%)	31(37.3%)/52(62.7%)	1.881	1.117–3.166	0.020*
	Homozygote	CC vs TT	32(24.4%)/99(75.6%)	14(21.2%)/52(78.8%)	1.201	0.589–2.447	0.722
	Additive	CC + TT vs TC	131(54.1%)/111(45.9%)	66(68.0%)/31(32.0%)	0.554	0.338–0.910	0.021*

Data are presented as n (%); CI, confidence interval; OR, odds ratio; *p* values were computed using the chi-square test, p < 0.05*;

Codominant model: GG vs AG vs AA; Allele model: G vs A; Dominant model: GG + AG vs AA; Recessive model: GG vs AG + AA;

Heterozygote model: AG vs AA; Homozygote model: GG vs AA. Additive: GG + AA vs AG

### Gender- specific variations in SNPs associated with schizophrenia

In terms of gender differences, we observed a higher susceptibility to schizophrenia in male cases compared to healthy males for rs3734442 and rs4431401. However, no significant disparity was found in the distribution of genotype and allele for the CD73 SNPs among female cases (Table 3). Among male patients, the G allele and AG heterozygote frequencies of rs3734442 were significantly lower than those in healthy males (OR = 0.452, 95% CI = 0.257–0.796, *p* = 0.007 for G/A; OR = 0.419, 95% CI = 0.209–0.841, *p* = 0.017 for AG/AA) (Table 3). The TC genotype frequency of rs4431401 was higher percentage among male cases with schizophrenia compared to healthy groups (OR = 2.570, 95% CI = 1.196–5.522, *p* = 0.015 for TC/TT; OR = 0.399, 95% CI = 0.194–0.821, *p* = 0.016 for CC + TT/TC) (Table 3). However, no statistically significant differences were observed in genotype and allele distribution within genders for other SNPs in CD73 (Supplementary Table S3).

**Table 3:** Genotypic and allelic distribution of the CD73 gene between schizophrenia patients and healthy controls of different genders

SNP	Gender	Genetic model	Genotype/allele	Schizophrenia patients	Healthy controls	OR	95% CI	P value
rs3734442	Male	Codominant	GG vs AG vs AA	1(0.7%)/39(27.5%)/102(71.8%)	2(4.3%)/21(45.7%)23(50.0%)	-	-	0.020*
		Allele	G vs A	41(14.4%)/243(85.6%)	25(27.2%)/67(72.8%)	0.452	0.257–0.796	0.007*
		Dominant	GG + AG vs AA	40(28.2%)/102(71.8%)	23(50.0%)/23(50.0%)	0.392	0.198–0.777	0.008*
		Recessive	GG vs AG + AA	1(0.7%)/141(99.3%)	2(4.3%)/44(95.7%)	0.156	0.014–1.762	0.149
		Heterozygote	AG vs AA	39(27.7%)/102(72.3%)	21(47.7%)/23(52.3%)	0.419	0.209–0.841	0.017*
		Homozygote	GG vs AA	1(1.0%)/102(99.0%)	2(8.0%)/23(92.0%)	0.113	0.010–1.297	0.097
		Additive	GG + AA vs AG	103(72.5%)/39(27.5%)	25(54.3%)/21(45.7%)	2.218	1.116–4.411	0.029*
	Female	Codominant	GG vs AG vs AA	3(3.1%)/37(37.8%)/58(59.2%)	4(7.8%)/20(39.2%)/27(52.9%)	-	-	0.366
		Allele	G vs A	43(21.9%)/153(78.1%)	28(27.5%)/74(72.5%)	0.743	0.428–1.289	0.317
		Dominant	GG + AG vs AA	40(40.8%)/58(59.2%)	24(47.1%)/27(52.9%)	0.776	0.392–1.534	0.490
		Recessive	GG vs AG + AA	3(3.1%)/95(96.9%)	4(7.8%)/47(92.2%)	0.371	0.080–1.726	0.231
		Heterozygote	AG vs AA	37(38.9%)/58(61.1%)	20(42.6%)/27(57.4%)	0.861	0.423–1.752	0.718
		Homozygote	GG vs AA	3(4.9%)/58(95.1%)	4(12.9%)/27(87.1%)	0.349	0.073–1.670	0.220
		Additive	GG + AA vs AG	61(62.2%)/37(37.8%)	31(60.8%)/20(39.2%)	1.064	0.531–2.131	1.000
rs4431401	Male	Codominant	CC vs TC vs TT	21(14.7%)/71(49.7%)/51(35.7%)	9(19.6%)/13(28.3%)/24(52.2%)	-	-	0.041*
		Allele	C vs T	113(39.5%)/173(60.5%)	31(33.7%)/61(66.3%)	1.285	0.785–2.104	0.327
		Dominant	CC + TC vs TT	92(64.3%)/51(35.7%)	22(47.8%)/24(52.2%)	1.968	1.005–3.854	0.057
		Recessive	CC vs TC + TT	21(14.7%)/122(85.3%)	9(19.6%)/37(80.4%)	0.708	0.299–1.678	0.487
		Heterozygote	TC vs TT	71(58.2%)/51(41.8%)	13(35.1%)/24(64.9%)	2.570	1.196–5.522	0.015*
		Homozygote	CC vs TT	21(29.2%)/51(70.8%)	9(27.3%)/24(72.7%)	1.098	0.438–2.753	1.000
		Additive	CC + TT vs TC	72(50.3%)/71(49.7%)	33(71.7%)/13(28.3%)	0.399	0.194–0.821	0.016*
	Female	Codominant	CC vs TC vs TT	11(11.1%)/40(40.4%)/48(48.5%)	5(9.8%)/18(35.3%)/28(54.9%)	-	-	0.799
		Allele	C vs T	62(31.3%)/136(68.7%)	28(28.0%)/72(72.0%)	1.172	0.690–1.991	0.595
		Dominant	CC + TC vs TT	51(51.5%)/48(48.5%)	23(45.1%)/28(54.9%)	1.293	0.657–2.548	0.494
		Recessive	CC vs TC + TT	11(11.1%)/88(88.9%)	5(9.8%)/46(90.2%)	1.150	0.377–3.509	1.000
		Heterozygote	TC vs TT	40(45.5%)/48(54.5%)	18(39.1%)/28(60.9%)	1.296	0.627–2.678	0.582
		Homozygote	CC vs TT	11(18.6%)/48(81.4%)	5(15.2%)/28(84.8%)	1.283	0.404–4.075	0.779
		Additive	CC + TT vs TC	59(59.6%)/40(40.4%)	33(64.7%)/18(35.3%)	0.805	0.399–1.621	0.598

Data are presented as n (%); CI, confidence interval; OR, odds ratio; *p* values were computed using the chi-square test, p < 0.05*

Codominant model: GG vs AG vs AA; Allele model: G vs A; Dominant model: GG + AG vs AA; Recessive model: GG vs AG + AA;

Heterozygote model: AG vs AA; Homozygote model: GG vs AA. Additive: GG + AA vs AG

### Family history of schizophrenia correlated to SNPs

Statistically significant variants in familial occurrence of schizophrenia were investigated among CD73 SNPs. Date show that individuals with a positive family history of schizophrenia who carry the C allele/TC genotype variant at CD73 rs4431401 have a higher percentage risk of developing schizophrenia than healthy subjects (*p* = 0.038; *p* = 0.007, respectively) (Table 4). Furthermore, patients with a family history showed a significantly higher prevalence of the G allele at rs9450282 compared to healthy individuals (*p* = 0.047) (Table 4). Conversely, the G allele of rs3734442 was found to be significantly lower in patients with a familial background of schizophrenia compared to healthy groups (*p* = 0.039) (Table 4). Interestingly, carriers of a variant allele at rs9444348 exhibited an increased genetic susceptibility to schizophrenia when compared to both individuals without a family history and healthy groups (*p* = 0.048, *p* = 0.031, respectively) (Table 4 and 5). No other correlations between family history and remaining SNPs were observed (Table 5 and Supplementary Table S4).

**Table 4:** Genotypic and allelic distribution of the CD73 gene between family history of schizophrenia cases and healthy controls

SNP	Genetic model	Genotype/allele	Family history of schizophrenia cases	Healthy controls	OR	95% CI	P value
rs9444348	Codominant	AA vs GA vs GG	10(16.7%)/31(51.7%)/19(31.7%)	13(13.4%)/35(36.1%)/49(50.5%)	-	-	0.066
	Allele	A vs G	51(42.5%)/69(57.5%)	61(31.4%)/133(68.6%)	1.612	1.005–2.584	0.053
	Dominant	AA + GA vs GG	41(68.3%)/19(31.7%)	48(49.5%)/49(50.5%)	2.203	1.123–4.322	0.031*
	Recessive	AA vs GA + GG	10(16.7%)/50(83.3%)	13(13.4%)/84(86.6%)	1.292	0.528–3.165	0.644
	Heterozygote	GA vs GG	31(62.0%)/19(38.0%)	35(41.7%)/49(58.3%)	2.284	1.115–4.679	0.032*
	Homozygote	AA vs GG	10(34.5%)/19(65.5%)	13(21.0%)/49(79.0%)	1.984	0.745–5.285	0.199
	Additive	AA + GG vs GA	29(48.3%)/31(51.7%)	62(63.9%)/35(36.1%)	0.528	0.275–1.016	0.068
rs9450282	Codominant	GG vs AG vs AA	14(23.3%)/31(51.7%)/15(25.0%)	16(16.5%)/41(42.3%)/40(41.2%)	-	-	0.114
	Allele	G vs A	59(49.2%)/61(50.8%)	73(37.6%)/121(62.4%)	1.603	1.011–2.542	0.047*
	Dominant	GG + AG vs AA	45(75.0%)/15(25.0%)	57(58.8%)/40(41.2%)	2.105	1.034–4.284	0.041*
	Recessive	GG vs AG + AA	14(23.3%)/46(76.7%)	16(16.5%)/81(83.5%)	1.541	0.690–3.441	0.303
	Heterozygote	AG vs AA	31(67.4%)/15(32.6%)	41(50.6%)/40(49.4%)	2.016	0.948–4.289	0.093
	Homozygote	GG vs AA	14(48.3%)/15(51.7%)	16(28.6%)//40(71.4%)	2.333	0.920–5.919	0.095
	Additive	GG + AA vs AG	29(48.3%)/31(51.7%)	56(57.7%)/41(42.3%)	0.685	0.359–1.308	0.323
rs4431401	Codominant	CC vs TC vs TT	10(16.7%)/31(51.7%)/19(31.7%)	14(14.4%)/31(32.0%)/52(53.6%)	-	-	0.021*
	Allele	C vs T	51(42.5%)/69(57.5%)	59(30.4%)/135(69.6%)	1.691	1.053–2.716	0.038*
	Dominant	CC + TC vs TT	41(68.3%)/19(31.7%)	45(46.4%)/52(53.6%)	2.494	1.270–4.896	0.008*
	Recessive	CC vs TC + TT	10(16.7%)/50(83.3%)	14(14.4%)/83(85.6%)	1.186	0.490–2.870	0.820
	Heterozygote	TC vs TT	31(62.0%)/19(38.0%)	31(37.3%)/52(62.7%)	2.737	1.327–5.644	0.007*
	Homozygote	CC vs TT	10(34.5%)/19(65.5%)	14(21.2%)/52(78.8%)	1.955	0.743–5.140	0.203
	Additive	CC + TT vs TC	29(48.3%)/31(51.7%)	66(68.0%)/31(32.0%)	0.439	0.227–0.852	0.019*
rs3734442	Codominant	GG vs AG vs AA	1(1.7%)/18(30.5%)/40(67.8%)	6(6.2%)/41(42.3%)/50(51.5%)	-	-	0.100
	Allele	G vs A	20(16.9%)/98(83.1%)	53(27.3%)/141(72.7%)	0.543	0.305–0.965	0.039*
	Dominant	GG + AG vs AA	19(32.2%)/40(67.8%)	47(48.5%)/50(51.5%)	0.505	0.257–0.993	0.066
	Recessive	GG vs AG + AA	1(1.7%)/58(98.3%)	6(6.2%)/91(93.8%)	0.261	0.031–2.228	0.255
	Heterozygote	AG vs AA	18(31.0%)/40(69.0%)	41(45.1%)/50(54.9%)	0.549	0.274–1.097	0.122
	Homozygote	GG vs AA	1(2.4%)/40(97.6%)	6(10.7%)/50(89.3%)	0.208	0.024–1.802	0.233
	Additive	GG + AA vs AG	41(69.5%)/18(30.5%)	56(57.7%)/41(42.3%)	1.668	0.841–3.309	0.174

Data are presented as n (%); CI, confidence interval; OR, odds ratio; *p* values were computed using the chi-square test, p < 0.05*

Codominant model: GG vs AG vs AA; Allele model: G vs A; Dominant model: GG + AG vs AA; Recessive model: GG vs AG + AA;

Heterozygote model: AG vs AA; Homozygote model: GG vs AA. Additive: GG + AA vs AG

**Table 5:** Genotypic and allelic distribution of the CD73 gene between family history of schizophrenia subjects and without family history of schizophrenia subjects

SNP	Genetic model	Genotype/allele	Family history of schizophrenia subjects	Without family history of schizophrenia subjects	OR	95% CI	P value
rs4431401	Codominant	CC vs TC vs TT	10(16.7%)/31(51.7%)/19(31.7%)	22(12.1%)/79(43.4%)/81(44.5%)	-	-	0.207
	Allele	C vs T	51(42.5%)/69(57.5%)	123(33.8%)/241(66.2%)	1.448	0.950–2.209	0.100
	Dominant	CC + TC vs TT	41(68.3%)/19(31.7%)	101(55.5%)/81(44.5%)	1.731	0.933–3.209	0.097
	Recessive	CC vs TC + TT	10(16.7%)/50(83.3%)	22(12.1%)/160(87.9%)	1.455	0.646–3.277	0.382
	Heterozygote	TC vs TT	31(62.0%)/19(38.0%)	79(49.4%)/81(50.6%)	1.673	0.874–3.204	0.145
	Homozygote	CC vs TT	10(34.5%)/19(65.5%)	22(21.4%)/81(78.6%)	1.938	0.789–4.762	0.219
	Additive	CC + TT vs TC	29(48.3%)/31(51.7%)	103(56.6%)/79(43.4%)	0.718	0.400–1.288	0.297
rs9444348	Codominant	AA vs GA vs GG	10(16.7%)/31(51.7%)/19(31.7%)	20(11.0%)/78(42.9%)/84(46.2%)	-	-	0.132
	Allele	A vs G	51(42.5%)/69(57.5%)	118(32.4%)/246(67.6%)	1.541	1.009–2.353	0.048*
	Dominant	AA + GA vs GG	41(68.3%)/19(31.7%)	98(53.8%)/84(46.2%)	1.850	0.998–3.428	0.052
	Recessive	AA vs GA + GG	10(16.7%)/50(83.3%)	20(11.0%)/162(89.0%)	1.620	0.712–3.688	0.262
	Heterozygote	GA vs GG	31(62.0%)/19(38.0%)	78(48.1%)/84(51.9%)	1.757	0.918–3.362	0.106
	Homozygote	AA vs GG	10(34.5%)/19(65.5%)	20(19.2%)/84(80.8%)	2.211	0.892–5.480	0.129
	Additive	AA + GG vs GA	29(48.3%)/31(51.7%)	104(57.1%)/78(42.9%)	0.702	0.391–1.260	0.295
rs3734442	Codominant	GG vs AG vs AA	1(1.7%)/18(30.5%)/40(67.8%)	4(2.2%)/57(31.5%)/120(66.3%)	-	-	0.949
	Allele	G vs A	20(16.9%)/98(83.1%)	65(18.0%)/297(82.0%)	0.932	0.538–1.617	0.890
	Dominant	GG + AG vs AA	19(32.2%)/40(67.8%)	61(33.7%)/120(66.3%)	0.934	0.499–1.749	0.875
	Recessive	GG vs AG + AA	1(1.7%)/58(98.3%)	4(2.2%)/177(97.8%)	0.763	0.084–6.964	1.000
	Heterozygote	AG vs AA	18(31.0%)/40(69.0%)	57(32.2%)/120(67.8%)	0.947	0.500–1.796	0.873
	Homozygote	GG vs AA	1(2.4%)/40(97.6%)	4(3.2%)/120(96.8%)	0.750	0.081–6.908	1.000
	Additive	GG + AA vs AG	41(69.5%)/18(30.5%)	124(68.5%)/57(31.5%)	1.047	0.554–1.979	1.000
rs6922	Codominant	GG vs GT vs TT	30(50.0%)/20(33.3%)/10(16.7%)	65(36.7%)/74(41.8%)/38(21.5%)	-	-	0.203
	Allele	G vs T	80(66.7%)/40(33.3%)	204(57.6%)/150 (42.4%)	1.471	0.953–2.270	0.086
	Dominant	GG + GT vs TT	50(83.3%)/10(16.7%)	139(78.5%)/38(21.5%)	1.367	0.634–2.946	0.464
	Recessive	GG vs GT + TT	30(50.0%)/30(50.0%)	65(36.7%)/112(63.3%)	1.723	0.954–3.112	0.093
	Heterozygote	GT vs TT	20(66.7%)/10(33.3%)	74(66.1%)/38(33.9%)	1.027	0.437–2.412	1.000
	Homozygote	GG vs TT	30(75.0%)/10(25.0%)	65(63.1%)/38(36.9%)	1.754	0.772–3.982	0.237
	Additive	GG + TT vs GT	40(66.7%)/20(33.3%)	103(58.2%)/74(41.8%)	1.437	0.777–2.656	0.286
rs2229523	Codominant	GG vs AG vs AA	30(50.0%)/20(33.3%)/10(16.7%)	66(36.5%)/77(42.5%)/38(21.0%)	-	-	0.185
	Allele	G vs A	80(66.7%)/40(33.3%)	209(57.7%)/153(42.3%)	1.464	0.950–2.258	0.087
	Dominant	GG + AG vs AA	50(83.3%)/10(16.7%)	143(79.0%)/38(21.0%)	1.329	0.617–2.862	0.577
	Recessive	GG vs AG + AA	30(50.0%)/30(50.0%)	66(36.5%)/115(63.5%)	1.742	0.966–3.142	0.069
	Heterozygote	AG vs AA	20(66.7%)/10(33.3%)	77(67.0%)/38(33.0%)	0.987	0.421–2.316	1.000
	Homozygote	GG vs AA	30(75.0%)/10(25.0%)	66(63.5%)/38(36.5%)	1.727	0.761–3.920	0.238
	Additive	GG + AA vs AG	40(66.7%)/20(33.3%)	104(57.5%)/77(42.5%)	1.481	0.803–2.732	0.227
rs4579322	Codominant	AA vs TA vs TT	29(49.2%)/20(33.9%)/10(16.9%)	68(37.8%)/68(37.8%)/44(24.4%)	-	-	0.274
	Allele	A vs T	78(66.1%)/40(33.9%)	204(56.7%)/156(43.3%)	1.491	0.966–2.303	0.084
	Dominant	AA + TA vs TT	49(83.1%)/10(16.9%)	136(75.6%)/44(24.4%)	1.585	0.741–3.391	0.283
	Recessive	AA vs TA + TT	29(49.2%)/30(50.8%)	68(37.8%)/112(62.2%)	1.592	0.880–2.880	0.130
	Heterozygote	TA vs TT	20(66.7%)/10(33.3%)	68(60.7%)/44(39.3%)	1.294	0.554–3.023	0.673
	Homozygote	AA vs TT	29(74.4%)/10(25.6%)	68(60.7%)/44(39.3%)	1.876	0.833–4.229	0.174
	Additive	AA + TT vs TA	39(66.1%)/20(33.9%)	112(62.2%)/68(37.8%)	1.184	0.638–2.195	0.643
rs9450282	Codominant	GG vs AG vs AA	14(23.3%)/31(51.7%)/15(25.0%)	31(17.1%)/84(46.4%)/66(36.5%)	-	-	0.223
	Allele	G vs A	59(49.2%)/61(50.8%)	146(40.3%)/216(59.7%)	1.431	0.945–2.167	0.110
	Dominant	GG + AG vs AA	45(75.0%)/15(25.0%)	115(63.5%)/66(36.5%)	1.722	0.892–3.325	0.116
	Recessive	GG vs AG + AA	14(23.3%)/46(76.7%)	31(17.1%)/150(82.9%)	1.473	0.722–3.002	0.339
	Heterozygote	AG vs AA	31(67.4%)/15(32.6%)	84(56.0%)/66(44.0%)	1.624	0.810–3.256	0.177
	Homozygote	GG vs AA	14(48.3%)/15(51.7%)	31(32.0%)/66(68.0%)	1.987	0.854–4.622	0.125
	Additive	GG + AA vs AG	29(48.3%)/31(51.7%)	97(53.6%)/84(46.4%)	0.810	0.452–1.454	0.551
rs2065114	Codominant	GG vs GA vs AA	29(48.3%)/22(36.7%)/9(15.0%)	68(37.6%)/75(41.4%)/38(21.0%)	-	-	0.306
	Allele	G vs A	80(66.7%)/40(33.3%)	211(58.3%)/151(41.7%)	1.431	0.928–2.207	0.108
	Dominant	GG + GA vs AA	51(85.0%)/9(15.0%)	143(79.0%)/38(21.0%)	1.506	0.681–3.331	0.352
	Recessive	GG vs GA + AA	29(48.3%)/31(51.7%)	68(37.6%)/113(62.4%)	1.555	0.863–2.801	0.172
	Heterozygote	GA vs AA	22(71.0%)/9(29.0%)	75(66.4%)/38(33.6%)	1.239	0.520–2.951	0.672
	Homozygote	GG vs AA	29(76.3%)/9(23.7%)	68(64.2%)/38(35.8%)	1.801	0.772–4.199	0.227
	Additive	GG + AA vs GA	38(63.3%)/22(36.7%)	106(58.6%)/75(41.4%)	1.222	0.669–2.233	0.547

Data are presented as n (%); CI, confidence interval; OR, odds ratio; *p* values were computed using the chi-square test, p < 0.05*;

Codominant model: GG vs AG vs AA; Allele model: G vs A; Dominant model: GG + AG vs AA; Recessive model: GG vs AG + AA;

Heterozygote model: AG vs AA; Homozygote model: GG vs AA. Additive: GG + AA vs AG

### CD73 Genetic variations associated with antipsychotic treatment

The administration of other antipsychotics did not reveal any potential association; however, it confirmed a correlation between the use of clozapine and specific SNPs in CD73 (Table 6 and 7; Supplementary Tables S5 and S6). Data demonstrates that individuals with schizophrenia who carry G allele of rs6922, rs2229523, and rs2065114 have a higher proportion of taking clozapine than using risperidone (OR = 1.816, 95% CI = 1.070–3.082, *p* = 0.035 for rs6922; OR = 1.742, 95% CI = 1.032–2.941, *p* = 0.049 for rs2229523; OR = 1.846, 95% CI = 1.096–3.110, *p* = 0.027 for rs2065114) (Table 6). Furthermore, there was a higher prevalence of the GG genotype of rs9444348 among cases using clozapine compared to those using sulpiride (OR = 0.352, 95% CI = 0.134–0.924, *p* = 0.048) (Table 7). These findings provide valuable insights into the genetic susceptibility for schizophrenia as well as potential targets for clinical therapeutics.

**Table 6:** Genotypic and allelic distribution of the CD73 gene between taking clozapine and taking risperidone

SNP	Genetic model	Genotype/allele	Clozapine	Risperidone	OR	95% CI	P value
rs6922	Codominant	GG vs GT vs TT	26(50.0%)/19(36.5%)/7(13.5%)	24(33.8%)/29(40.8%)18(25.4%)	-	-	0.133
	Allele	G vs T	71(68.3%)/33(31.7%)	77(54.2%)/65(45.8%)	1.816	1.070–3.082	0.035*
	Dominant	GG + GT vs TT	45(86.5%)/7(13.5%)	53(74.6%)/18(25.4%)	2.183	0.837–5.697	0.118
	Recessive	GG vs GT + TT	26(50.0%)/26(50.0%)	24(33.8%)/47(66.2%)	1.958	0.941–4.076	0.094
	Heterozygote	GT vs TT	19(73.1%)7(26.9%)	29(61.7%)18(38.3%)	1.685	0.591–4.801	0.441
	Homozygote	GG vs TT	26(78.8%)7(21.2%)	24(57.1%)18(42.9%)	2.786	0.990–7.837	0.083
	Additive	GG + TT vs GT	33(63.5%)/19(36.5%)	42(59.2%)/29(40.8%)	1.199	0.574–2.505	0.709
rs2229523	Codominant	GG vs AG vs AA	26(49.1%)/20(37.7%)7(13.2%)	25(34.7%)/29(40.3%)/18(25.0%)	-	-	0.171
	Allele	G vs A	72(67.9%)/34(32.1%)	79(54.9%)/65(45.1%)	1.742	1.032–2.941	0.049*
	Dominant	GG + AG vs AA	46(86.8%)/7(13.2%)	54(75.0%)/18(25.0%)	2.190	0.841–5.707	0.118
	Recessive	GG vs AG + AA	26(49.1%)/27(50.9%)	25(34.7%)/47(65.3%)	1.810	0.877–3.737	0.141
	Heterozygote	AG vs AA	20(74.1%)/7(25.9%)	29(61.7%)/18(38.3%)	1.773	0.625–5.030	0.318
	Homozygote	GG vs AA	26(78.8%)7(21.2%)	25(58.1%)/18(41.9%)	2.674	0.953–7.501	0.084
	Additive	GG + AA vs AG	33(62.3%)/20(37.7%)	43(59.7%)/29(40.3%)	1.113	0.537–2.305	0.854
rs2065114	Codominant	GG vs GA vs AA	26(49.1%)/20(37.7%)/7(13.2%)	24(32.9%)/30(41.1%)/19(26.0%)	-	-	0.116
	Allele	G vs A	72(67.9%)/34(32.1%)	78(53.4%)/68(46.6%)	1.846	1.096–3.110	0.027*
	Dominant	GG + GA vs AA	46(86.8%)/7(13.2%)	54(74.0%)/19(26.0%)	2.312	0.893–5.988	0.118
	Recessive	GG vs GA + AA	26(49.1%)/27(50.9%)	24(32.9%)/49(67.1%)	1.966	0.950–4.067	0.096
	Heterozygote	GA vs AA	20(74.1%)/7(25.9%)	30(61.2%)/19(38.8%)	1.810	0.643–5.094	0.318
	Homozygote	GG vs AA	26(78.8%)/7(21.2%)	24(55.8%)/19(44.2%)	2.940	1.051–8.228	0.051
	Additive	GG + AA vs GA	33(62.3%)/20(37.7%)	43(58.9%)/30(41.1%)	1.151	0.557–2.377	0.717

Data are presented as n (%); CI, confidence interval; OR, odds ratio; *p* values were computed using the chi-square test, p < 0.05*;

Codominant model: GG vs AG vs AA; Allele model: G vs A; Dominant model: GG + AG vs AA; Recessive model: GG vs AG + AA;

Heterozygote model: AG vs AA; Homozygote model: GG vs AA. Additive: GG + AA vs AG

**Table 7:** Genotypic and allelic distribution of the CD73 gene between taking clozapine and taking sulpiride

SNP	Genetic model	Genotype/allele	Clozapine	Sulpiride	OR	95% CI	P value
rs9444348	Codominant	AA vs GA vs GG	5(9.4%)/23(43.4%)/25(47.2%)	2(9.1%)/11(50.0%)/9(40.9%)	-	-	0.930
	Allele	A vs G	33(31.1%)/73(68.9%)	15(34.9%)/28(65.1%)	0.844	0.399–1.786	0.701
	Dominant	AA + GA vs GG	28(33.7%)/25(66.3%)	13(59.1%)/9(40.9%)	0.352	0.134–0.924	0.048*
	Recessive	AA vs GA + GG	5(9.4%)/48(90.6%)	2(9.1%)/20(90.9%)	1.042	0.186–5.822	1.000
	Heterozygote	GA vs GG	23(47.9%)/25(52.1%)	11(55.0%)/9(45.0%)	0.753	0.264–2.145	0.791
	Homozygote	AA vs GG	5(16.7%)/25(83.3%)	2(18.2%)/9(81.8%)	0.900	0.148–5.489	1.000
	Additive	AA + GG vs GA	30(56.6%)/23(43.4%)	11(50.0%)/11(50.0%)	1.304	0.481–3.534	0.620
rs6922	Codominant	GG vs GT vs TT	26(50.0%)/19(36.5%)/7(13.5%)	9(42.9%)/8(38.1%)/4(19.0%)	-	-	0.782
	Allele	G vs T	71(68.3%)/33(31.7%)	26(61.9%)/16(38.1%)	1.324	0.627–2.795	0.562
	Dominant	GG + GT vs TT	45(86.5%)/7(13.5%)	17(81.0%)/4(19.0%)	1.513	0.392–5.830	0.719
	Recessive	GG vs GT + TT	26(50.0%)/26(50.0%)	9(42.9%)/12(57.1%)	1.333	0.480–3.701	0.615
	Heterozygote	GT vs TT	19(73.1%)7(26.9%)	8(66.7%)/4(33.3%)	1.357	0.309–5.964	0.714
	Homozygote	GG vs TT	26(78.8%)7(21.2%)	9(69.2%)/4(30.8%)	1.651	0.390–6.992	0.702
	Additive	GG + TT vs GT	33(63.5%)/19(36.5%)	13(61.9%)/8(38.1%)	1.069	0.375–3.042	1.000
rs2229523	Codominant	GG vs AG vs AA	26(49.1%)/20(37.7%)7(13.2%)	9(40.9%)/9(40.9%)/4(18.2%)	-	-	0.834
	Allele	G vs A	72(67.9%)/34(32.1%)	27(61.4%)/17(38.6%)	1.333	0.642–2.770	0.454
	Dominant	GG + AG vs AA	46(86.8%)/7(13.2%)	18(81.8%)/4(18.2%)	1.460	0.381–5.599	0.721
	Recessive	GG vs AG + AA	26(49.1%)/27(50.9%)	9(40.9%)/13(59.1%)	1.391	0.509–3.804	0.615
	Heterozygote	AG vs AA	20(74.1%)/7(25.9%)	9(69.2%)/4(30.8%)	1.270	0.295–5.461	1.000
	Homozygote	GG vs AA	26(78.8%)7(21.2%)	9(69.2%)/4(30.8%)	1.651	0.390–6.992	0.702
	Additive	GG + AA vs AG	33(62.3%)/20(37.7%)	13(59.1%)/9(40.9%)	1.142	0.414–3.153	1.000
rs4579322	Codominant	AA vs TA vs TT	26(50.0%)/16(30.8%)/10(19.2%)	8(38.1%)/9(42.9%)/4(19.0%)	-	-	0.623
	Allele	A vs T	68(65.4%)/36(34.6%)	25(59.5%)/17(40.5%)	1.284	0.615–2.683	0.570
	Dominant	AA + TA vs TT	42(80.8%)/10(19.2%)	17(81.0%)/4(19.0%)	0.988	0.272–3.587	1.000
	Recessive	AA vs TA + TT	26(50.0%)/26(50.0%)	8(38.1%)/13(61.9%)	1.625	0.577–4.574	0.441
	Heterozygote	TA vs TT	16(61.5%)/10(38.5%)	9(69.2%)/4(30.8%)	0.711	0.172–2.937	0.733
	Homozygote	AA vs TT	26(72.2%)/10(27.8%)	8(66.7%)/4(33.3%)	1.300	0.319–5.295	1.000
	Additive	AA + TT vs TA	36(69.2%)/16(30.8%)	12(57.1%)/9(42.9%)	1.688	0.593–4.802	0.415
rs9450282	Codominant	GG vs AG vs AA	9(17.0%)/29(54.7%)/15(28.3%)	3(14.3%)/12(57.1%)/6(28.6%)	-	-	1.000
	Allele	G vs A	47(44.3%)/59(55.7%)	18(42.9%)/24(57.1%)	1.062	0.516–2.185	1.000
	Dominant	GG + AG vs AA	38(71.7%)/15(28.3%)	15(71.4%)/6(28.6%)	1.013	0.331–3.105	1.000
	Recessive	GG vs AG + AA	9(17.0%)/44(83.0%)	3(14.3%)/18(85.7%)	1.227	0.298–5.062	1.000
	Heterozygote	AG vs AA	29(65.9%)/15(34.1%)	12(66.7%)6(33.3%)	0.967	0.303–3.088	1.000
	Homozygote	GG vs AA	9(37.5%)/15(62.5%)	3(33.3%)/6(66.7%)	1.200	0.239–6.025	1.000
	Additive	GG + AA vs AG	24(45.3%)/29(54.7%)	9(42.9%)/12(57.1%)	1.103	0.398–3.059	1.000
rs4431401	Codominant	CC vs TC vs TT	6(11.3%)/24(45.3%)/23(43.4%)	2(9.1%)/11(50.0%)/9(40.9%)	-	-	0.936
	Allele	C vs T	36(34.0%)/70(66.0%)	15(34.1%)/29(65.9%)	0.994	0.474–2.088	1.000
	Dominant	CC + TC vs TT	30(56.6%)23(43.4%)	13(59.1%)/9(40.9%)	0.903	0.329–2.476	1.000
	Recessive	CC vs TC + TT	6(11.3%)/47(88.7%)	2(9.1%)/20(90.9%)	1.277	0.237–6.875	1.000
	Heterozygote	TC vs TT	24(51.1%)/23(48.9%)	11(55.0%)/9(45.0%)	0.854	0.299–2.440	0.796
	Homozygote	CC vs TT	6(20.7%)/23(79.3%)	2(18.2%)/9(81.8%)	1.174	0.199–6.935	1.000
	Additive	CC + TT vs TC	29(54.7%)/24(45.3%)	11(50.0%)/11(50.0%)	1.208	0.447–3.270	0.801
rs2065114	Codominant	GG vs GA vs AA	26(49.1%)/20(37.7%)/7(13.2%)	8(38.1%)/9(42.9%)/4(19.0%)	-	-	0.647
	Allele	G vs A	72(67.9%)/34(32.1%)	25(59.5%)/17(40.5%)	1.440	0.688–3.015	0.344
	Dominant	GG + GA vs AA	46(86.8%)/7(13.2%)	17(81.0%)4(19.0%)	1.546	0.401–5.956	0.718
	Recessive	GG vs GA + AA	26(49.1%)/27(50.9%)	8(38.1%)/13(61.9%)	1.565	0.557–4.393	0.446
	Heterozygote	GA vs AA	20(74.1%)/7(25.9%)	9(69.2%)/4(30.8%)	1.270	0.295–5.461	1.000
	Homozygote	GG vs AA	26(78.8%)/7(21.2%)	8(66.7%)/4(33.3%)	1.857	0.430–8.012	0.448
	Additive	GG + AA vs GA	33(62.3%)/20(37.7%)	12(57.1%)/9(42.9%)	1.238	0.443–3.457	0.793

Data are presented as n (%); CI, confidence interval; OR, odds ratio; *p* values were computed using the chi-square test, p < 0.05*;

Codominant model: GG vs AG vs AA; Allele model: G vs A; Dominant model: GG + AG vs AA; Recessive model: GG vs AG + AA;

Heterozygote model: AG vs AA; Homozygote model: GG vs AA. Additive: GG + AA vs AG

## Discussion

We verified whether genetic variations in CD73 are connected with the risk of schizophrenia by investigating a total of thirty single nucleotide polymorphisms (SNPs). Importantly, we discovered that two SNPs, rs3734442 and rs4431401, remained significantly associated with male cases even after adjusting for gender. Additionally, we observed an association between certain SNPs in CD73 and the use of clozapine as an antipsychotic medication, including rs6922, rs2229523, rs2065114, and rs9444348. Furthermore, considering potential family history factors, we identified several SNPs in CD73 (rs9444348, rs9450282, rs4431401, and rs3734442) that were linked to the genetic risk of schizophrenia. Notably, this is the first clinical evaluation to data examining how variations in CD73/ecto-5’-nuclotidase influence the risk of schizophrenia. Our data show a heightened genetic susceptibility to schizophrenia related to CD73. Moreover, the findings from our study support the hypothesis that genetic variations in CD73 are associated with schizophrenia and suggest a potential role in its onset.

CD73/ecto-5’-nucleotidase plays a crucial role in metabolic processes contributing to extracellular adenosine levels. Furthermore, numerous studies consistently report significantly reduced extracellular adenosine levels during the pathophysiological processes of schizophrenia, which are associated with hyperdopaminergic states and dysregulated glutamatergic transmission. These factors may contribute to the susceptibility to schizophrenia [23, 24]. Although there is limited literature on CD73, current evidence suggests that disruptions in CD73 function can impact the adenosine cycle and potentially influence the development of schizophrenia. Therefore, our results show that genetic variations may alter ecto-5’-nucleotidase function, leading to decreased extracellular adenosine levels and potentially influencing the occurrence of schizophrenia.

Several studies have reported a connection between rs4431401 and urinary system disorders, such as childhood nephrotic syndrome (NS) and calcium uremic arteriolopathy (CUA). These studies found that the rare alleles of rs4431401 were associated with a higher risk of steroid resistance and calciphylaxis in CUA patients [25, 26]. Recent research has also demonstrated a strong correlation between the variant allele rs4431401 and an increased risk of epilepsy [27]. Unfortunately, we could not find any comparable literature on rs3734442 when searching PubMed for English publications. We suspect that this may be due to differences in samples size, which will require further validation of rs3734442 in future experiments. Nonetheless, our study suggests that the polymorphisms rs3734442 may be associated with an increased risk of schizophrenia, although extensive literature research is needed to support this finding in the future. Importantly, both rs4431401 and rs3734442 were identified as significant risk factors for schizophrenia based on our experimental data, indicating their potential role in this disorder. Given CD73's regulatory function in the adenosine cycle pathway, these findings are not surprising; however, further investigations are required to determine if there are any effects on CD73 gene expression resulting from variations explored in this present study on schizophrenia.

The prevalence of schizophrenia exhibits gender disparities, characterized by a higher incidence in male patients. Most studies have found that males with schizophrenia experience an earlier age of onset and more severe negative symptoms than females [28, 29]. Sex differences in schizophrenia may be attributed to genetic factors, brain structure variations, the disease process itself, and hormonal disparities [30]. However, emerging research suggests that estrogen plays a vital role in these gender differences [31]. Animal studies also indicate that estrogens influence neurite growth and synapse formation while regulating neurotransmitters systems such as dopamine [29]. Furthermore, rodent studies demonstrate the involvement of adenosine in 17β-estradiol (E2)-mediated hippocampal synaptic rearrangement. E2 not only reduces CD73 activity in the hippocampal synaptic region of male rats but also increases it in female rats [32, 33].

In our study, we observed a significant association between genetic variants of CD73 and gender differences (rs3734442 and rs4431401). A separate study on epilepsy patients reported that the variant rs4431401 exhibited a significant associated with both female and male cases compared to healthy controls [27]. However, our data indicate that the rs4431401 variant is specifically associated with schizophrenia in males, while no significant difference was observed in females. Furthermore, there is limited literature available regarding the impact of genetic variations in rs3734442 on gender differences. Despite this lack of knowledge, it has been found that genetic variations of CD73 are linked to male patients. These gender disparities may be attributed to estrogen metabolism, adenosine levels, and hormone-gene interactions, suggesting novel biological pathways relevant to schizophrenia.

Another intriguing finding of our study was the observed relationship between gene variations in CD73 single nucleotide polymorphisms (SNPs) and a positive family history of schizophrenia among subjects in subgroup analysis. The presence of a family history has been proposed as an important risk factor for schizophrenia [34]. Previous studies have suggested that individuals homozygous for CD73 variants rs9444348 and rs9450282 were associated with an increased incidence and seizure frequency of epilepsy following traumatic brain injury (TBI) [35]. Rs9444348 is located in the promoter flanking region of the CD73 gene, which could a potentially contribute to genetic variation related to a family history of schizophrenia. Early genomic study on human heart have indicated that rs4431401 can regulate the expression of small nucleolar RNA host gene 5, while this particular gene primarily regulates rRNA modification [36, 37].

Therefore, our findings suggest that the CD73 variant may have a significant impact on the presence of schizophrenia within family history. However, further investigations are required to explore the potential association between CD73 gene expression and familial susceptibility based on our dataset. In the future studies, we aim to investigate target genes across three generations with a family history of schizophrenia as well as among relatives affected by this disorder.

Ecto-5’-nucleotidase plays an important role in the regulation of adenosine within the purinergic pathway, while adenosine involvement has been reported in schizophrenia [23]. A compelling study has indicated an upregulation of A2ARs in the hippocampus and striatum of individuals with schizophrenia compared to controls, suggesting that this increase in A2AR expression may be attributed to compensatory responses to low adenosine levels or antipsychotic treatment [38, 39]. Moreover, adjunctive therapy with an adenosine modulator has demonstrated significant efficacy for patients with schizophrenia, particularly in ameliorating positive symptoms [40].

In the current study, significant differences were observed in SNPs associated with antipsychotic use. Specifically, rs6922, rs2229523, rs9450282, and rs2065114 SNPs were found to be significantly associated with patients taking clozapine than those taking other antipsychotics. Previous research has linked the genotype of rs6922 to treatment outcomes in colorectal cancer patients receiving anti-vascular endothelial growth factor (VEGF) targeted therapy [41]. Additionally, the CD73 gene variant rs2229523 has been associated with a higher probability of longer median overall survival in metastatic colorectal cancer cases following bevacizumab chemotherapy [42]. These findings suggest that genetic variations in the CD73 gene may have a critical impact on drug treatment response and prognosis. However, whether there is a real association between taking clozapine and variations in the CD73 gene remains uncertain; therefore, it is unclear if our results can be widely applied in clinical practice. Moreover, future studies on schizophrenia drug therapy should incorporate analysis of genetic polymorphisms related to ecto-5’-nucleotidase/CD73.

We must acknowledge the limitations when accounting for the current study. On the one hand, we only analyzed the population in southwestern Chin because representation from other regions of the country is lacking. Further studies should involve patients from the different regions. On the other hand, the sample sizes of some subgroups are very small after stratification, and different stratifications yield leads to different positive SNP results. Therefore, in the future, we will increase the number of controls, and optimizing the stratification analyses. Despite these limitations, our findings provide evidence for the involvement of CD73 polymorphisms in schizophrenia.

## Supplementary Information

Below is the link to the electronic supplementary material.

Supplementary file1 (DOCX 19 KB)

Supplementary file2 (DOCX 29 KB)

Supplementary file3 (DOCX 39 KB)

Supplementary file4 (DOCX 23 KB)

Supplementary file5 (DOCX 29 KB)

Supplementary file6 (DOCX 24 KB)

## Data Availability

The datasets used and analysed are available from the corresponding authors upon reasonable request.
