# Supplementary material for: CD73 polymorphisms are associated with schizophrenia
Source: Purinergic Signal. 2024 May 17;21(4):695–707. doi: 10.1007/s11302-024-10004-3 (PMC12454215; doi:10.1007/s11302-024-10004-3)
Supplement: Supplementary file 1 — Supplementary file1 (DOCX 19 KB) [file 11302_2024_10004_MOESM1_ESM.docx]

Table 1 Hardy-Weinberg equilibrium of CD73 SNPs site

| SNP | Group | Gene type (frequency) | P |
| --- | --- | --- | --- |
| rs3734442 | Schizophrenia patients | 4(1.7%)/75(31.3%)/161(67.1%) | 0.566 |
|  | Healthy controls | 6(6.2%)/41(42.3%)/50(51.5%) | 0.949 |
| rs4431401 | Schizophrenia patients | 32(13.2%)/111(45.9%)/99(40.9%) | 0.999 |
|  | Healthy controls | 14(14.4%)/31(32.0%)/52(53.6%) | 0.250 |
| rs9444348 | Schizophrenia patients | 30(12.4%)/110(45.5%)/102(42.1%) | 1.000 |
|  | Healthy controls | 13(13.4%)/35(36.1%)/49(50.5%) | 0.611 |
| rs6922 | Schizophrenia patients | 94(39.7%)/95(40.1%)/48(20.3%) | 0.217 |
|  | Healthy controls | 42(43.3%)/42(43.3%)/13(13.4%) | 0.960 |
| rs2229523 | Schizophrenia patients | 95(39.4%)/98(40.7%)/48(19.9%) | 0.229 |
|  | Healthy controls | 43(44.8%)/40(41.7%)/13(13.5%) | 0.844 |
| rs4579322 | Schizophrenia patients | 96(40.2%)/89(37.2%)/54(22.6%) | 0.044 |
|  | Healthy controls | 41(42.7%)/37(38.5%)/18(18.8%) | 0.492 |
| rs9450282 | Schizophrenia patients | 45(18.7%)/116(48.1%)/80(33.2%) | 0.985 |
|  | Healthy controls | 16(16.5%)/41(42.3%)/40(41.2%) | 0.807 |
| rs2065114 | Schizophrenia patients | 96(39.8%)/98(40.7%)/47(19.5%) | 0.234 |
|  | Healthy controls | 42(43.3%)/40(41.2%)/15(15.5%) | 0.803 |

* The degree of discrepancy between the observed and theoretical values is indicated by P.

* P＜0.05 indicates statistical significance.
