# Supplementary material for: CD73 polymorphisms are associated with schizophrenia
Source: Purinergic Signal. 2024 May 17;21(4):695–707. doi: 10.1007/s11302-024-10004-3 (PMC12454215; doi:10.1007/s11302-024-10004-3)
Supplement: Supplementary file 2 — Supplementary file2 (DOCX 29 KB) [file 11302_2024_10004_MOESM2_ESM.docx]

Table2 Genotypic and allelic distribution of the CD73 gene between schizophrenia patients and healthy controls

| SNP | Genetic model | Genotype/allele | Schizophrenia patients | Healthy controls | OR | 95% CI | P value |
| --- | --- | --- | --- | --- | --- | --- | --- |
| rs9444348 | Codominant | AA vs GA vs GG | 30(12.4%)/110(45.5%)/102(42.1%) | 13(13.4%)/35(36.1%)/49(50.5%) | - | - | 0.279 |
|  | Allele | A vs G | 110(25.9%)/314(74.1%) | 61(31.4%)/133(68.6%) | 0.764 | 0.526-1.109 | 0.175 |
|  | Dominant | AA+GA vs GG | 140(57.9%)/102(42.1%) | 48(49.5%)/49(50.5%) | 1.401 | 0.873-2.248 | 0.184 |
|  | Recessive | AA vs GA+GG | 30(12.4%)/212(87.6%) | 13(13.4%)/84(86.6%) | 0.914 | 0.455-1.838 | 0.857 |
|  | Heterozygote | GA vs GG | 110(51.9%)/102(48.1%) | 35(41.7%)/49(58.3%) | 1.510 | 0.906-2.516 | 0.123 |
|  | Homozygote | AA vs GG | 30(22.7%)/102(77.3%) | 13(21.0%)/49(79.0%) | 1.109 | 0.532-2.311 | 0.854 |
|  | Additive | AA+GG vs GA | 132(54.5%)/110(45.5%) | 62(63.9%)/35(36.1%) | 0.677 | 0.417-1.101 | 0.145 |
| rs6922 | Codominant | GG vs GT vs TT | 94(39.7%)/95(40.1%)/48(20.3%) | 42(43.3%)/42(43.3%)/13(13.4%) | - | - | 0.344 |
|  | Allele | G vs T | 283(59.7%)/191(40.3%) | 126(64.9%)/68(35.1%) | 0.800 | 0.565-1.132 | 0.221 |
|  | Dominant | GG+GT vs TT | 189(79.7%)/48(20.3%) | 84(86.6%)/13(13.4%) | 0.609 | 0.314-1.184 | 0.162 |
|  | Recessive | GG vs GT+TT | 94(39.7%)/143(60.3%) | 42(43.3%)/55(56.7%) | 0.861 | 0.533-1.389 | 0.542 |
|  | Heterozygote | GT vs TT | 95(66.4%)/48(33.6%) | 42(76.4%)/13(23.6%) | 0.613 | 0.300-1.249 | 0.229 |
|  | Homozygote | GG vs TT | 94(66.2%)/48(33.8%) | 42(76.4%)/13(23.6%) | 0.606 | 0.297-1.236 | 0.175 |
|  | Additive | GG+TT vs GT | 142(59.9%)/95(40.1%) | 55(56.7%)/42(43.3%) | 1.141 | 0.707-1.842 | 0.625 |
| rs2229523 | Codominant | GG vs AG vs AA | 95(39.4%)/98(40.7%)/48(19.9%) | 43(44.8%)/40(41.7%)/13(13.5%) | - | - | 0.366 |
|  | Allele | G vs A | 288(59.8%)/194(40.2%) | 128(66.0%)/66(34.0%) | 0.765 | 0.540-1.084 | 0.138 |
|  | Dominant | GG+AG vs AA | 193(80.1%)/48(19.9%) | 83(86.5%)/13(13.5%) | 0.630 | 0.324-1.224 | 0.210 |
|  | Recessive | GG vs AG+AA | 95(39.4%)/146(60.6%) | 43(44.8%)/53(55.2%) | 0.802 | 0.497-1.294 | 0.392 |
|  | Heterozygote | AG vs AA | 98(67.1%)/48(32.9%) | 40(75.5%)/13(24.5%) | 0.664 | 0.325-1.356 | 0.299 |
|  | Homozygote | GG vs AA | 95(66.4%)/48(33.6%) | 43(76.8%)/13(23.2%) | 0.598 | 0.294-1.218 | 0.174 |
|  | Additive | GG+AA vs AG | 143(59.3%)/98(40.7%) | 56(58.3%)/40(41.7%) | 1.042 | 0.645-1.685 | 0.903 |
| rs4579322 | Codominant | AA vs TA vs TT | 96(40.2%)/89(37.2%)/54(22.6%) | 41(42.7%)/37(38.5%)/18(18.8%) | - | - | 0.747 |
|  | Allele | A vs T | 281(58.8%)/197(41.2%) | 118(61.8%)/73(38.2%) | 0.882 | 0.626-1.245 | 0.487 |
|  | Dominant | AA+TA vs TT | 185(77.4%)/54(22.6%) | 78(81.3%)/18(18.8%) | 0.791 | 0.436-1.434 | 0.466 |
|  | Recessive | AA vs TA+TT | 96(40.2%)/143(59.8%) | 41(42.7%)/55(57.3%) | 0.901 | 0.557-1.455 | 0.713 |
|  | Heterozygote | TA vs TT | 89(62.2%)/54(37.8%) | 37(67.3%)/18(32.7%) | 0.802 | 0.416-1.547 | 0.621 |
|  | Homozygote | AA vs TT | 96(64.0%)/54(36.0%) | 41(69.5%)/18(30.5%) | 0.780 | 0.409-1.490 | 0.519 |
|  | Additive | AA+TT vs TA | 150(62.8%)/89(37.2%) | 59(61.5%)/37(38.5%) | 1.057 | 0.649-1.721 | 0.901 |
| rs9450282 | Codominant | GG vs AG vs AA | 45(18.7%)/116(48.1%)/80(33.2%) | 16(16.5%)/41(42.3%)/40(41.2%) | - | - | 0.401 |
|  | Allele | G vs A | 206(42.7%)/276(57.3%) | 73(37.6%)/121(62.4%) | 1.237 | 0.879-1.742 | 0.228 |
|  | Dominant | GG+AG vs AA | 161(66.8%)/80(33.2%) | 57(58.8%)/40(41.2%) | 1.412 | 0.869-2.294 | 0.169 |
|  | Recessive | GG vs AG+AA | 45(18.7%)/196(81.3%) | 16(16.5%)/81(83.5%) | 1.162 | 0.621-2.175 | 0.645 |
|  | Heterozygote | AG vs AA | 116(59.2%)/80(40.8%) | 41(50.6%)/40(49.4%) | 1.415 | 0.841-2.381 | 0.230 |
|  | Homozygote | GG vs AA | 45(36.0%)/80(64.0%) | 16(28.6%)//40(71.4%) | 1.406 | 0.709-2.790 | 0.396 |
|  | Additive | GG+AA vs AG | 125(51.9%)/116(48.1%) | 56(57.7%)/41(42.3%) | 0.789 | 0.490-1.269 | 0.338 |
| rs2065114 | Codominant | GG vs GA vs AA | 96(39.8%)/98(40.7%)/47(19.5%) | 42(43.3%)/40(41.2%)/15(15.5%) | - | - | 0.667 |
|  | Allele | G vs A | 290(60.2%)/192(39.8%) | 124(63.9%)/70(36.1%) | 0.853 | 0.604-1.204 | 0.384 |
|  | Dominant | GG+GA vs AA | 194(80.5%)/47(19.5%) | 82(84.5%)/15(15.5%) | 0.755 | 0.400-1.426 | 0.439 |
|  | Recessive | GG vs GA+AA | 96(39.8%)/145(60.2%) | 42(43.3%)/55(56.7%) | 0.867 | 0.538-1.397 | 0.625 |
|  | Heterozygote | GA vs AA | 98(67.6%)/47(32.4%) | 40(72.7%)/15(27.3%) | 0.782 | 0.393-1.555 | 0.500 |
|  | Homozygote | GG vs AA | 96(67.1%)/47(32.9%) | 42(73.7%)/15(26.3%) | 0.729 | 0.368-1.447 | 0.401 |
|  | Additive | GG+AA vs GA | 143(59.3%)/98(40.7%) | 57(58.8%)/40(41.2%) | 1.024 | 0.634-1.653 | 1.000 |

Data are presented as n (%); CI, confidence interval; OR, odds ratio; *p* values were computed using the chi-square test, p＜0.05*;

Codominant model: GG vs AG vs AA; Allele model: G vs A; Dominant model: GG+AG vs AA; Recessive model: GG vs AG + AA;

Heterozygote model: AG vs AA; Homozygote model: GG vs AA. Additive: GG+AA vs AG.
