# Supplementary material for: CD73 polymorphisms are associated with schizophrenia
Source: Purinergic Signal. 2024 May 17;21(4):695–707. doi: 10.1007/s11302-024-10004-3 (PMC12454215; doi:10.1007/s11302-024-10004-3)
Supplement: Supplementary file 3 — Supplementary file3 (DOCX 39 KB) [file 11302_2024_10004_MOESM3_ESM.docx]

Table 3 Genotypic and allelic distribution of the CD73 gene between schizophrenia patients and healthy controls of different genders

| SNP | Gender | Genetic model | Genotype/allele | Schizophrenia patients | Healthy controls | OR | 95% CI | P value |
| --- | --- | --- | --- | --- | --- | --- | --- | --- |
| rs4579322 | Male | Codominant | AA vs TA vs TT | 57(40.4%)/55(39.0%)/29(20.6%) | 23(51.1%)/13(28.9%)/9(20.0%) | - | - | 0.386 |
|  |  | Allele | A vs T | 169(59.9%)/113(40.1%) | 59(65.6%)/31(34.4%) | 0.786 | 0.479-1.290 | 0.385 |
|  |  | dominant | AA+TA vs TT | 112(79.4%)/29(20.6%) | 36(80.0%)/9(20.0%) | 0.966 | 0.418-2.229 | 1.000 |
|  |  | Recessive | AA vs TA+TT | 57(40.4%)/84(59.6%) | 23(51.1%)/22(48.9%) | 0.649 | 0.331-1.274 | 0.229 |
|  |  | Heterozygote | TA vs TT | 55(65.5%)/29(34.5%) | 13(59.1%)/9(40.9%) | 1.313 | 0.502-3.435 | 0.622 |
|  |  | Homozygote | AA vs TT | 57(66.3%)/29(33.7%) | 23(71.9%)/9(28.1%) | 0.769 | 0.316-1.875 | 0.660 |
|  |  | Additive | AA+TT vs TA | 86(61.0%)/55(39.0%) | 32(71.1%)/13(28.9%) | 0.635 | 0.307-1.316 | 0.286 |
|  | Female | Codominant | AA vs TA vs TT | 40(40.8%)/33(33.7%)25(25.5%) | 18(35.3%)/24(47.1%)/9(17.6%) | - | - | 0.252 |
|  |  | Allele | A vs T | 113(57.7%)/83(42.3%) | 60(58.8%)/42(41.2%) | 0.953 | 0.586-1.549 | 0.902 |
|  |  | dominant | AA+TA vs TT | 73(74.5%)/25(25.5%) | 42(82.4%)/9(17.6%) | 0.626 | 0.267-1.466 | 0.311 |
|  |  | Recessive | AA vs TA+TT | 40(40.8%)/58(59.2%) | 18(35.3%)/33(64.7%) | 1.264 | 0.627-2.550 | 0.596 |
|  |  | Heterozygote | TA vs TT | 33(56.9%)/25(43.1%) | 24(72.7%)/9(27.3%) | 0.495 | 0.196-1.249 | 0.177 |
|  |  | Homozygote | AA vs TT | 40(61.5%)/25(38.5%) | 18(66.7%)/9(33.3%) | 0.800 | 0.311-2.055 | 0.813 |
|  |  | Additive | AA+TT vs TA | 65(66.3%)/33(33.7%) | 27(52.9%)/24(47.1%) | 1.751 | 0.877-3.495 | 0.155 |
| rs9444348 | Male | Codominant | AA vs GA vs GG | 20(14.0%)/69(48.3%)/54(37.8%) | 9(19.6%)/15(32.6%)/22(47.8%) | - | - | 0.192 |
|  |  | Allele | A vs G | 109(38.1%)/177(61.9%) | 33(35.9%)/59(64.1%) | 1.101 | 0.676-1.794 | 0.713 |
|  |  | dominant | AA+GA vs GG | 89(62.2%)/54(37.8%) | 24(52.2%)/22(47.8%) | 1.511 | 0.773-2.953 | 0.232 |
|  |  | Recessive | AA vs GA+GG | 20(14.0%)/123(86.0%) | 9(19.6%)/37(80.4%) | 0.668 | 0.281-1.593 | 0.481 |
|  |  | Heterozygote | GA vs GG | 69(56.1%)/54(43.9%) | 15(40.5%)/22(59.5%) | 1.874 | 0.888-3.954 | 0.133 |
|  |  | Homozygote | AA vs GG | 20(27.0%)/54(73.0%) | 9(29.0%)/22(71.0%) | 0.905 | 0.357-2.294 | 1.000 |
|  |  | Additive | AA+GG vs GA | 74(51.7%)/69(48.3%) | 31(67.4%)/15(32.6%) | 0.519 | 0.258-1.043 | 0.087 |
|  | Female | Codominant | AA vs GA vs GG | 10(10.1%)/41(41.4%)/48(48.5%) | 4(7.8%)/20(39.2%)/27(52.9%) | - | - | 0.846 |
|  |  | Allele | A vs G | 61(30.8%)/137(69.2%) | 28(27.5%)/74(72.5%) | 1.177 | 0.693-1.998 | 0.595 |
|  |  | dominant | AA+GA vs GG | 51(51.5%)/48(48.5%) | 24(47.1%)/27(52.9%) | 1.195 | 0.608-2.351 | 0.730 |
|  |  | Recessive | AA vs GA+GG | 10(10.1%)/89(89.9%) | 4(7.8%)/47(92.2%) | 1.320 | 0.393-4.437 | 0.773 |
|  |  | Heterozygote | GA vs GG | 41(46.1%)/48(53.9%) | 20(42.6%)/27(57.4%) | 1.153 | 0.565-2.352 | 0.720 |
|  |  | Homozygote | AA vs GG | 10(17.2%)/48(82.8%) | 4(12.9%)/27(87.1%) | 1.406 | 0.402-4.917 | 0.763 |
|  |  | Additive | AA+GG vs GA | 58(58.6%)/41(41.4%) | 31(60.8%)/20(39.2%) | 0.913 | 0.458-1.819 | 0.862 |
| rs9450282 | Male | Codominant | GG vs AG vs AA | 30(21.1%)/75(52.8%)/37(26.1%) | 10(21.7%)/19(41.3%)/17(37.0%) | - | - | 0.326 |
|  |  | Allele | G vs A | 135(47.5%)/149(52.5%) | 39(42.4%)/53(57.6%) | 1.231 | 0.766-1.979 | 0.402 |
|  |  | dominant | GG+AG vs AA | 105(73.9%)/37(26.1%) | 29(63.0%)/17(37.0%) | 1.664 | 0.821-3.371 | 0.190 |
|  |  | Recessive | GG vs AG+AA | 30(21.1%)/112(78.9%) | 10(21.7%)/36(78.3%) | 0.964 | 0.430-2.164 | 1.000 |
|  |  | Heterozygote | AG vs AA | 75(67.0%)/37(33.0%) | 19(52.8%)/17(47.2%) | 1.814 | 0.845-3.892 | 0.163 |
|  |  | Homozygote | GG vs AA | 30(44.8%)/37(55.2%) | 10(37.0%)/17(63.0%) | 1.378 | 0.551-3.450 | 0.645 |
|  |  | Additive | GG+AA vs AG | 67(47.2%)/75(52.8%) | 27(58.7%)19(41.3%) | 0.629 | 0.321-1.232 | 0.235 |
|  | Female | Codominant | GG vs AG vs AA | 15(15.2%)/41(41.4%)/43(43.4%) | 6(11.8%)/22(43.1%)/23(45.1%) | - | - | 0.893 |
|  |  | Allele | G vs A | 71(35.9%)/127(64.1%) | 34(33.3%)/68(66.7%) | 1.118 | 0.676-1.850 | 0.703 |
|  |  | dominant | GG+AG vs AA | 56(56.6%)/43(43.4%) | 28(54.9%)/23(45.1%) | 1.070 | 0.542-2.111 | 0.864 |
|  |  | Recessive | GG vs AG+AA | 15(15.2%)/84(84.8%) | 6(11.8%)/45(88.2%) | 1.339 | 0.486-3.691 | 0.629 |
|  |  | Heterozygote | AG vs AA | 41(48.8%)/43(51.2%) | 22(48.9%)23(51.1%) | 0.997 | 0.483-2.057 | 1.000 |
|  |  | Homozygote | GG vs AA | 15(25.9%)/43(74.1%) | 6(20.7%)/23(79.3%) | 1.337 | 0.457-3.913 | 0.791 |
|  |  | Additive | GG+AA vs AG | 58(58.6%)/41(41.4%) | 29(56.9%)/22(43.1%) | 1.073 | 0.542-2.125 | 0.863 |
| rs2065114 | Male | Codominant | GG vs GA vs AA | 57(40.1%)/61(43.0%)/24(16.9%) | 23(50.0%)/16(34.8%)/7(15.2%) | - | - | 0.515 |
|  |  | Allele | G vs A | 175(61.6%)/109(38.4%) | 62(67.4%)/30(32.6%) | 0.777 | 0.473-1.277 | 0.324 |
|  |  | dominant | GG+GA vs AA | 118(83.1%)/24(16.9%) | 39(84.8%)/7(15.2%) | 0.882 | 0.353-2.207 | 0.825 |
|  |  | Recessive | GG vs GA+AA | 57(40.1%)/85(59.9%) | 23(50.0%)/23(50.0%) | 0.671 | 0.344-1.308 | 0.303 |
|  |  | Heterozygote | GA vs AA | 61(71.8%)/24(28.2%) | 16(69.6%)/7(30.4%) | 1.112 | 0.407-3.041 | 1.000 |
|  |  | Homozygote | GG vs AA | 57(70.4%)/24(29.6%) | 23(76.7%)/7(23.3%) | 0.723 | 0.274-1.909 | 0.636 |
|  |  | Additive | GG+AA vs GA | 81(57.0%)/61(43.0%) | 30(65.2%)/16(34.8%) | 0.708 | 0.355-1.414 | 0.390 |
|  | Female | Codominant | GG vs GA vs AA | 40(40.4%)/36(36.4%)/23(23.2%) | 19(37.3%)/24(47.1%)/8(15.7%) | - | - | 0.392 |
|  |  | Allele | G vs A | 116(58.6%)/82(41.4%) | 62(60.8%)/40(39.2%) | 0.913 | 0.560-1.487 | 0.804 |
|  |  | dominant | GG+GA vs AA | 76(76.8%)/23(23.2%) | 43(84.3%)8(15.7%) | 0.615 | 0.253-1.493 | 0.299 |
|  |  | Recessive | GG vs GA+AA | 40(40.4%)/59(59.6%) | 19(37.3%)/32(62.7%) | 1.142 | 0.570-2.288 | 0.728 |
|  |  | Heterozygote | GA vs AA | 36(61.0%)/23(39.0%) | 24(75.0%)/8(25.0%) | 0.522 | 0.201-1.357 | 0.247 |
|  |  | Homozygote | GG vs AA | 40(63.5%)/23(36.5%) | 19(70.4%)8(29.6%) | 0.732 | 0.277-1.936 | 0.631 |
|  |  | Additive | GG+AA vs GA | 63(63.6%)/36(36.4%) | 27(52.9%)/24(47.1%) | 1.556 | 0.784-3.088 | 0.222 |
| rs2229523 | Male | Codominant | GG vs AG vs AA | 57(40.1%)/60(42.3%)/25(17.6%) | 24(52.2%)/15(32.6%)/7(15.2%) | - | - | 0.357 |
|  |  | Allele | G vs A | 174(61.3%)/110(38.7%) | 63(68.5%)/29(31.5%) | 0.728 | 0.441-1.201 | 0.218 |
|  |  | dominant | GG+AG vs AA | 117(82.4%)/25(17.6%) | 39(84.8%)/7(15.2%) | 0.840 | 0.337-2.093 | 0.824 |
|  |  | Recessive | GG vs AG+AA | 57(40.1%)/85(59.9%) | 24(52.2%)/22(47.8%) | 0.615 | 0.315-1.200 | 0.172 |
|  |  | Heterozygote | AG vs AA | 60(70.6%)/25(29.4%) | 15(68.2%)/7(31.8%) | 1.120 | 0.407-3.079 | 1.000 |
|  |  | Homozygote | GG vs AA | 57(69.5%)/25(30.5%) | 24(77.4%)/7(22.6%) | 0.665 | 0.254-1.744 | 0.487 |
|  |  | Additive | GG+AA vs AG | 82(57.7%)/60(42.3%) | 31(67.4%)/15(32.6%) | 0.661 | 0.328-1.333 | 0.300 |
|  | Female | Codominant | GG vs AG vs AA | 39(39.4%)/37(37.4%)/23(23.2%) | 19(38.0%)/25(50.0%)/6(12.0%) | - | - | 0.190 |
|  |  | Allele | G vs A | 115(58.1%)/83(41.9%) | 63(63.0%)/37(37.0%) | 0.814 | 0.496-1.334 | 0.454 |
|  |  | dominant | GG+AG vs AA | 76(76.8%)/23(23.2%) | 44(88.0%)/6(12.0%) | 0.451 | 0.170-1.191 | 0.127 |
|  |  | Recessive | GG vs AG+AA | 39(39.4%)/60(60.6%) | 19(38.0%)/31(62.0%) | 1.061 | 0.527-2.134 | 1.000 |
|  |  | Heterozygote | AG vs AA | 37(61.7%)/23(38.3%) | 25(80.6%)/6(19.4%) | 0.386 | 0.138-1.083 | 0.096 |
|  |  | Homozygote | GG vs AA | 39(62.9%)/23(37.1%) | 19(76.0%)/6(24.0%) | 0.535 | 0.187-1.534 | 0.318 |
|  |  | Additive | GG+AA vs AG | 62(62.6%)/37(37.4%) | 25(50.0%)/25(50.0%) | 1.676 | 0.842-3.334 | 0.161 |
| rs6922 | Male | Codominant | GG vs GT vs TT | 57(40.4%)/59(41.8%)/25(17.7%) | 23(50.0%)/16(34.8%)/7(15.2%) | - | - | 0.530 |
|  |  | Allele | G vs T | 173(61.3%)/109(38.7%) | 62(67.4%)/30(32.6%) | 0.768 | 0.467-1.263 | 0.322 |
|  |  | dominant | GG+GT vs TT | 116(82.3%)/25(17.7%) | 39(84.8%)/7(15.2%) | 0.833 | 0.334-2.076 | 0.823 |
|  |  | Recessive | GG vs GT+TT | 57(40.4%)/84(59.6%) | 23(50.0%)/23(50.0%) | 0.679 | 0.348-1.324 | 0.304 |
|  |  | Heterozygote | GT vs TT | 59(70.2%)/25(29.8%) | 16(69.6%)/7(30.4%) | 1.033 | 0.378-2.817 | 1.000 |
|  |  | Homozygote | GG vs TT | 57(69.5%)/25(30.5%) | 23(76.7%)/7(23.3%) | 0.694 | 0.264-1.827 | 0.491 |
|  |  | Additive | GG+TT vs GT | 82(58.2%)/59(41.8%) | 30(65.2%)/16(34.8%) | 0.741 | 0.371-1.482 | 0.489 |
|  | Female | Codominant | GG vs GT vs TT | 38(39.6%)/35(36.5%)/23(24.0%) | 19(37.2%)/26(51.0%)/6(11.8%) | - | - | 0.125 |
|  |  | Allele | G vs T | 111(57.8%)/81(42.2%) | 64(62.7%)/38(37.3%) | 0.814 | 0.497-1.332 | 0.455 |
|  |  | dominant | GG+GT vs TT | 73(76.0%)/23(24.0%) | 45(88.2%)/6(11.8%) | 0.423 | 0.160-1.119 | 0.086 |
|  |  | Recessive | GG vs GT+TT | 38(39.6%)/58(60.4%) | 19(37.3%)/32(62.7%) | 1.103 | 0.548-2.221 | 0.860 |
|  |  | Heterozygote | GT vs TT | 35(60.3%)/23(39.7%) | 26(81.3%)/6(18.8%) | 0.351 | 0.125-0.985 | 0.059 |
|  |  | Homozygote | GG vs TT | 38(62.3%)/23(37.7%) | 19(76.0%)/6(24.0%) | 0.522 | 0.182-1.497 | 0.316 |
|  |  | Additive | GG+TT vs GT | 61(63.5%)/35(36.5%) | 25(49.0%)/26(51.0%) | 1.813 | 0.910-3.609 | 0.114 |

Data are presented as n (%); CI, confidence interval; OR, odds ratio; *p* values were computed using the chi-square test, p＜0.05*;

Codominant model: GG vs AG vs AA; Allele model: G vs A; Dominant model: GG+AG vs AA; Recessive model: GG vs AG + AA;

Heterozygote model: AG vs AA; Homozygote model: GG vs AA. Additive: GG+AA vs AG.
