# Supplementary material for: CD73 polymorphisms are associated with schizophrenia
Source: Purinergic Signal. 2024 May 17;21(4):695–707. doi: 10.1007/s11302-024-10004-3 (PMC12454215; doi:10.1007/s11302-024-10004-3)
Supplement: Supplementary file 4 — Supplementary file4 (DOCX 23 KB) [file 11302_2024_10004_MOESM4_ESM.docx]

Table 4 Genotypic and allelic distribution of the CD73 gene between family history of schizophrenia cases and healthy controls

| SNP | Genetic model | Genotype/allele | Family history of schizophrenia cases | Healthy controls | OR | 95% CI | P value |
| --- | --- | --- | --- | --- | --- | --- | --- |
| rs2229523 | Codominant | GG vs AG vs AA | 30(50.0%)/20(33.3%)/10(16.7%) | 43(44.8%)/40(41.7%)/13(13.5%) | - | - | 0.580 |
|  | Allele | G vs A | 80(66.7%)/40(33.3%) | 128(66.0%)/66(34.0%) | 1.031 | 0.637-1.670 | 0.903 |
|  | Dominant | GG+AG vs AA | 50(83.3%)/10(16.7%) | 83(86.5%)/13(13.5%) | 0.783 | 0.320-1.918 | 0.646 |
|  | Recessive | GG vs AG+AA | 30(50.0%)/30(50.0%) | 43(44.8%)/53(55.2%) | 1.233 | 0.646-2.353 | 0.621 |
|  | Heterozygote | AG vs AA | 20(66.7%)/10(33.3%) | 40(75.5%)/13(24.5%) | 0.650 | 0.243-1.738 | 0.448 |
|  | Homozygote | GG vs AA | 30(75.0%)/10(25.0%) | 43(76.8%)/13(23.2%) | 0.907 | 0.352-2.338 | 1.000 |
|  | Additive | GG+AA vs AG | 40(66.7%)/20(33.3%) | 56(58.3%)/40(41.7%) | 1.429 | 0.729-2.800 | 0.316 |
| rs4579322 | Codominant | AA vs TA vs TT | 29(49.2%)/20(33.9%)/10(16.9%) | 41(42.7%)/37(38.5%)/18(18.8%) | - | - | 0.743 |
|  | Allele | A vs T | 78(66.1%)/40(33.9%) | 118(61.8%)/73(38.2%) | 1.206 | 0.746-1.950 | 0.468 |
|  | Dominant | AA+TA vs TT | 49(83.1%)/10(16.9%) | 78(81.3%)/18(18.8%) | 1.131 | 0.483-2.650 | 0.833 |
|  | Recessive | AA vs TA+TT | 29(49.2%)/30(50.8%) | 41(42.7%)/55(57.3%) | 1.297 | 0.676-2.487 | 0.507 |
|  | Heterozygote | TA vs TT | 20(66.7%)/10(33.3%) | 37(67.3%)/18(32.7%) | 0.973 | 0.378-2.504 | 1.000 |
|  | Homozygote | AA vs TT | 29(74.4%)/10(25.6%) | 41(69.5%)/18(30.5%) | 1.273 | 0.514-3.155 | 0.654 |
|  | Additive | AA+TT vs TA | 39(66.1%)/20(33.9%) | 59(61.5%)/37(38.5%) | 1.223 | 0.621-2.409 | 0.609 |
| rs6922 | Codominant | GG vs GT vs TT | 30(50.0%)/20(33.3%)/10(16.7%) | 42(43.3%)/42(43.3%)/13(13.4%) | - | - | 0.458 |
|  | Allele | G vs T | 80(66.7%)/40(33.3%) | 126(64.9%)/68(35.1%) | 1.079 | 0.667-1.745 | 0.807 |
|  | Dominant | GG+GT vs TT | 50(83.3%)/10(16.7%) | 84(86.6%)/13(13.4%) | 0.774 | 0.316-1.895 | 0.644 |
|  | Recessive | GG vs GT+TT | 30(50.0%)/30(50.0%) | 42(43.3%)/55(56.7%) | 1.310 | 0.686-2.499 | 0.510 |
|  | Heterozygote | GT vs TT | 20(66.7%)/10(33.3%) | 42(76.4%)/13(23.6%) | 0.619 | 0.232-1.652 | 0.444 |
|  | Homozygote | GG vs TT | 30(75.0%)/10(25.0%) | 42(76.4%)/13(23.6%) | 0.929 | 0.360-2.397 | 1.000 |
|  | Additive | GG+TT vs GT | 40(66.7%)/20(33.3%) | 55(56.7%)/42(43.3%) | 1.527 | 0.781-2.986 | 0.242 |
| rs2065114 | Codominant | GG vs GA vs AA | 29(48.3%)/22(36.7%)/9(15.0%) | 42(43.3%)/40(41.2%)/15(15.5%) | - | - | 0.815 |
|  | Allele | G vs A | 80(66.7%)/40(33.3%) | 124(63.9%)/70(36.1%) | 1.129 | 0.699-1.824 | 0.629 |
|  | Dominant | GG+GA vs AA | 51(85.0%)/9(15.0%) | 82(84.5%)/15(15.5%) | 1.037 | 0.423-2.543 | 1.000 |
|  | Recessive | GG vs GA+AA | 29(48.3%)/31(51.7%) | 42(43.3%)/55(56.7%) | 1.225 | 0.642-2.338 | 0.621 |
|  | Heterozygote | GA vs AA | 22(71.0%)/9(29.0%) | 40(72.7%)/15(27.3%) | 0.917 | 0.345-2.434 | 1.000 |
|  | Homozygote | GG vs AA | 29(76.3%)/9(23.7%) | 42(73.7%)/15(26.3%) | 1.151 | 0.444-2.982 | 0.814 |
|  | Additive | GG+AA vs GA | 38(63.3%)/22(36.7%) | 57(58.8%)/40(41.2%) | 1.212 | 0.625-2.351 | 0.617 |

Data are presented as n (%); CI, confidence interval; OR, odds ratio; *p* values were computed using the chi-square test, p＜0.05*;

Codominant model: GG vs AG vs AA; Allele model: G vs A; Dominant model: GG+AG vs AA; Recessive model: GG vs AG + AA;

Heterozygote model: AG vs AA; Homozygote model: GG vs AA. Additive: GG+AA vs AG.
