# Supplementary material for: CD73 polymorphisms are associated with schizophrenia
Source: Purinergic Signal. 2024 May 17;21(4):695–707. doi: 10.1007/s11302-024-10004-3 (PMC12454215; doi:10.1007/s11302-024-10004-3)
Supplement: Supplementary file 5 — Supplementary file5 (DOCX 29 KB) [file 11302_2024_10004_MOESM5_ESM.docx]

Table 5 Genotypic and allelic distribution of the CD73 gene between taking risperidone and taking sulpiride

| SNP | Genetic model | Genotype/allele | Risperidone | Sulpiride | OR | 95% CI | P value |
| --- | --- | --- | --- | --- | --- | --- | --- |
| rs9444348 | Codominant | AA vs GA vs GG | 9(12.3%)/34(46.6%)/30(41.1%) | 2(9.1%)/11(50.0%)/9(40.9%) | - | - | 0.946 |
|  | Allele | A vs G | 52(35.6%)/94(64.4%) | 15(34.9%)/28(65.1%) | 1.033 | 0.506-2.106 | 1.000 |
|  | Dominant | AA+GA vs GG | 43(58.9%)/30(41.1%) | 13(59.1%)/9(40.9%) | 0.992 | 0.376-2.616 | 1.000 |
|  | Recessive | AA vs GA+GG | 9(12.3%)/64(87.7%) | 2(9.1%)/20(90.9%) | 1.406 | 0.280-7.052 | 0.735 |
|  | Heterozygote | GA vs GG | 34(53.1%)/30(46.9%) | 11(55.0%)/9(45.0%) | 0.927 | 0.338-2.542 | 1.000 |
|  | Homozygote | AA vs GG | 9(23.1%)/30(76.9%) | 2(18.2%)/9(81.8%) | 1.350 | 0.246-7.417 | 1.000 |
|  | Additive | AA+GG vs GA | 39(53.4%)/34(46.6%) | 11(50.0%)/11(50.0%) | 1.147 | 0.442-2.978 | 0.812 |
| rs6922 | Codominant | GG vs GT vs TT | 24(33.8%)/29(40.8%)18(25.4%) | 9(42.9%)/8(38.1%)/4(19.0%) | - | - | 0.777 |
|  | Allele | G vs T | 77(54.2%)/65(45.8%) | 26(61.9%)/16(38.1%) | 0.729 | 0.360-1.475 | 0.479 |
|  | Dominant | GG+GT vs TT | 53(74.6%)/18(25.4%) | 17(81.0%)/4(19.0%) | 0.693 | 0.206-2.331 | 0.584 |
|  | Recessive | GG vs GT+TT | 24(33.8%)/47(66.2%) | 9(42.9%)/12(57.1%) | 0.681 | 0.252-1.840 | 0.605 |
|  | Heterozygote | GT vs TT | 29(61.7%)18(38.3%) | 8(66.7%)/4(33.3%) | 0.806 | 0.212-3.066 | 1.000 |
|  | Homozygote | GG vs TT | 24(57.1%)18(42.9%) | 9(69.2%)/4(30.8%) | 0.593 | 0.157-2.234 | 0.528 |
|  | Additive | GG+TT vs GT | 42(59.2%)/29(40.8%) | 13(61.9%)/8(38.1%) | 0.891 | 0.328-2.422 | 1.000 |
| rs2229523 | Codominant | GG vs AG vs AA | 25(34.7%)/29(40.3%)/18(25.0%) | 9(40.9%)/9(40.9%)/4(18.2%) | - | - | 0.782 |
|  | Allele | G vs A | 79(54.9%)/65(45.1%) | 27(61.4%)/17(38.6%) | 0.765 | 0.384-1.526 | 0.490 |
|  | Dominant | GG+AG vs AA | 54(75.0%)/18(25.0%) | 18(81.8%)/4(18.2%) | 0.667 | 0.199-2.230 | 0.580 |
|  | Recessive | GG vs AG+AA | 25(34.7%)/47(65.3%) | 9(40.9%)/13(59.1%) | 0.768 | 0.289-2.044 | 0.620 |
|  | Heterozygote | AG vs AA | 29(61.7%)/18(38.3%) | 9(69.2%)/4(30.8%) | 0.716 | 0.192-2.671 | 0.751 |
|  | Homozygote | GG vs AA | 25(58.1%)/18(41.9%) | 9(69.2%)/4(30.8%) | 0.617 | 0.164-2.321 | 0.535 |
|  | Additive | GG+AA vs AG | 43(59.7%)/29(40.3%) | 13(59.1%)/9(40.9%) | 1.027 | 0.388-2.712 | 1.000 |
| rs4579322 | Codominant | AA vs TA vs TT | 24(32.9%)/29(39.7%)/20(27.4%) | 8(38.1%)/9(42.9%)/4(19.0%) | - | - | 0.779 |
|  | Allele | A vs T | 77(52.7%)/69(47.3%) | 25(59.5%)/17(40.5%) | 0.759 | 0.378-1.523 | 0.485 |
|  | Dominant | AA+TA vs TT | 53(72.6%)/20(27.4%) | 17(81.0%)/4(19.0%) | 0.624 | 0.187-2.080 | 0.575 |
|  | Recessive | AA vs TA+TT | 24(32.9%)/49(67.1%) | 8(38.1%)/13(61.9%) | 0.796 | 0.291-2.179 | 0.794 |
|  | Heterozygote | TA vs TT | 29(59.2%)/20(40.8%) | 9(69.2%)/4(30.8%) | 0.644 | 0.174-2.384 | 0.545 |
|  | Homozygote | AA vs TT | 24(54.5%)/20(45.5%) | 8(66.7%)/4(33.3%) | 0.600 | 0.157-2.289 | 0.525 |
|  | Additive | AA+TT vs TA | 44(60.3%)/29(39.7%) | 12(57.1%)/9(42.9%) | 1.138 | 0.426-3.042 | 0.806 |
| rs9450282 | Codominant | GG vs AG vs AA | 9(17.0%)/29(54.7%)/15(28.3%) | 3(14.3%)/12(57.1%)/6(28.6%) | - | - | 1.000 |
|  | Allele | G vs A | 47(44.3%)/59(55.7%) | 18(42.9%)/24(57.1%) | 1.062 | 0.516-2.185 | 1.000 |
|  | Dominant | GG+AG vs AA | 38(71.7%)/15(28.3%) | 15(71.4%)/6(28.6%) | 1.013 | 0.331-3.105 | 1.000 |
|  | Recessive | GG vs AG+AA | 9(17.0%)/44(83.0%) | 3(14.3%)/18(85.7%) | 1.227 | 0.298-5.062 | 1.000 |
|  | Heterozygote | AG vs AA | 29(65.9%)/15(34.1%) | 12(66.7%)6(33.3%) | 0.967 | 0.303-3.088 | 1.000 |
|  | Homozygote | GG vs AA | 9(37.5%)/15(62.5%) | 3(33.3%)/6(66.7%) | 1.200 | 0.239-6.025 | 1.000 |
|  | Additive | GG+AA vs AG | 24(45.3%)/29(54.7%) | 9(42.9%)/12(57.1%) | 1.103 | 0.398-3.059 | 1.000 |
| rs4431401 | Codominant | CC vs TC vs TT | 6(11.3%)/24(45.3%)/23(43.4%) | 2(9.1%)/11(50.0%)/9(40.9%) | - | - | 0.936 |
|  | Allele | C vs T | 36(34.0%)/70(66.0%) | 15(34.1%)/29(65.9%) | 0.994 | 0.474-2.088 | 1.000 |
|  | Dominant | CC+TC vs TT | 30(56.6%)23(43.4%) | 13(59.1%)/9(40.9%) | 0.903 | 0.329-2.476 | 1.000 |
|  | Recessive | CC vs TC+TT | 6(11.3%)/47(88.7%) | 2(9.1%)/20(90.9%) | 1.277 | 0.237-6.875 | 1.000 |
|  | Heterozygote | TC vs TT | 24(51.1%)/23(48.9%) | 11(55.0%)/9(45.0%) | 0.854 | 0.299-2.440 | 0.796 |
|  | Homozygote | CC vs TT | 6(20.7%)/23(79.3%) | 2(18.2%)/9(81.8%) | 1.174 | 0.199-6.935 | 1.000 |
|  | Additive | CC+TT vs TC | 29(54.7%)/24(45.3%) | 11(50.0%)/11(50.0%) | 1.208 | 0.447-3.270 | 0.801 |
| rs2065114 | Codominant | GG vs GA vs AA | 24(32.9%)/30(41.1%)/19(26.0%) | 8(38.1%)/9(42.9%)/4(19.0%) | - | - | 0.819 |
|  | Allele | G vs A | 78(53.4%)/68(46.6%) | 25(59.5%)/17(40.5%) | 0.780 | 0.389-1.566 | 0.598 |
|  | Dominant | GG+GA vs AA | 54(74.0%)/19(26.0%) | 17(81.0%)4(19.0%) | 0.669 | 0.200-2.238 | 0.580 |
|  | Recessive | GG vs GA+AA | 24(32.9%)/49(67.1%) | 8(38.1%)/13(61.9%) | 0.796 | 0.291-2.179 | 0.794 |
|  | Heterozygote | GA vs AA | 30(61.2%)/19(38.8%) | 9(69.2%)/4(30.8%) | 0.702 | 0.189-2.602 | 0.751 |
|  | Homozygote | GG vs AA | 24(55.8%)/19(44.2%) | 8(66.7%)/4(33.3%) | 0.632 | 0.165-2.419 | 0.533 |
|  | Additive | GG+AA vs GA | 43(58.9%)/30(41.1%) | 12(57.1%)/9(42.9%) | 1.075 | 0.403-2.870 | 1.000 |

Data are presented as n (%); CI, confidence interval; OR, odds ratio; *p* values were computed using the chi-square test, p＜0.05*;

Codominant model: GG vs AG vs AA; Allele model: G vs A; Dominant model: GG+AG vs AA; Recessive model: GG vs AG + AA;

Heterozygote model: AG vs AA; Homozygote model: GG vs AA. Additive: GG+AA vs AG.
