# Supplementary material for: CD73 polymorphisms are associated with schizophrenia
Source: Purinergic Signal. 2024 May 17;21(4):695–707. doi: 10.1007/s11302-024-10004-3 (PMC12454215; doi:10.1007/s11302-024-10004-3)
Supplement: Supplementary file 6 — Supplementary file6 (DOCX 24 KB) [file 11302_2024_10004_MOESM6_ESM.docx]

Table 6 Genotypic and allelic distribution of the CD73 gene between taking clozapine and taking risperidone

| SNP | Genetic model | Genotype/allele | Clozapine | Risperidone | OR | 95% CI | P value |
| --- | --- | --- | --- | --- | --- | --- | --- |
| rs9444348 | Codominant | AA vs GA vs GG | 5(9.4%)/23(43.4%)/25(47.2%) | 9(12.3%)/34(46.6%)/30(41.1%) | - | - | 0.762 |
|  | Allele | A vs G | 33(31.1%)/73(68.9%) | 52(35.6%)/94(64.4%) | 0.817 | 0.480-1.392 | 0.501 |
|  | Dominant | AA+GA vs GG | 28(52.8%)/25(47.2%) | 43(58.9%)/30(41.1%) | 0.781 | 0.383-1.594 | 0.586 |
|  | Recessive | AA vs GA+GG | 5(9.4%)/48(90.6%) | 9(12.3%)/64(87.7%) | 0.741 | 0.233-2.352 | 0.776 |
|  | Heterozygote | GA vs GG | 23(47.9%)/25(52.1%) | 34(53.1%)/30(46.9%) | 0.812 | 0.384-1.718 | 0.703 |
|  | Homozygote | AA vs GG | 5(16.7%)/25(83.3%) | 9(23.1%)/30(76.9%) | 0.667 | 0.198-2.247 | 0.561 |
|  | Additive | AA+GG vs GA | 30(56.6%)/23(43.4%) | 39(53.4%)/34(46.6%) | 1.137 | 0.558-2.317 | 0.856 |
| rs9450282 | Codominant | GG vs AG vs AA | 9(17.0%)/29(54.7%)/15(28.3%) | 13(17.8%)/35(47.9%)/25(34.2%) | - | - | 0.761 |
|  | Allele | G vs A | 47(44.3%)/59(55.7%) | 61(41.8%)/85(58.2%) | 1.110 | 0.670-1.839 | 0.701 |
|  | Dominant | GG+AG vs AA | 38(71.7%)/15(28.3%) | 48(65.8%)/25(34.2%) | 1.319 | 0.612-2.846 | 0.562 |
|  | Recessive | GG vs AG+AA | 9(17.0%)/44(83.0%) | 13(17.8%)/60(82.2%) | 0.944 | 0.371-2.404 | 1.000 |
|  | Heterozygote | AG vs AA | 29(65.9%)/15(34.1%) | 35(58.3%)/25(41.7%) | 1.381 | 0.616-3.096 | 0.541 |
|  | Homozygote | GG vs AA | 9(37.5%)/15(62.5%) | 13(34.2%)/25(65.8%) | 1.154 | 0.398-3.344 | 1.000 |
|  | Additive | GG+AA vs AG | 24(45.3%)/29(54.7%) | 38(52.1%)/35(47.9%) | 0.762 | 0.375-1.550 | 0.476 |
| rs4431401 | Codominant | CC vs TC vs TT | 6(11.3%)/24(45.3%)/23(43.4%) | 9(12.3%)/36(49.3%)/28(38.4%) | - | - | 0.875 |
|  | Allele | C vs T | 36(34.0%)/70(66.0%) | 54(37.0%)/92(63.0%) | 0.876 | 0.519-1.480 | 0.690 |
|  | Dominant | CC+TC vs TT | 30(56.6%)23(43.4%) | 45(61.6%)/28(38.4%) | 0.812 | 0.395-1.666 | 0.586 |
|  | Recessive | CC vs TC+TT | 6(11.3%)/47(88.7%) | 9(12.3%)/64(87.7%) | 0.908 | 0.302-2.726 | 1.000 |
|  | Heterozygote | TC vs TT | 24(51.1%)/23(48.9%) | 36(56.3%)/28(43.8%) | 0.812 | 0.381-1.728 | 0.700 |
|  | Homozygote | CC vs TT | 6(20.7%)/23(79.3%) | 9(24.3%)/28(75.7%) | 0.812 | 0.252-2.618 | 0.775 |
|  | Additive | CC+TT vs TC | 29(54.7%)/24(45.3%) | 37(50.7%)/36(49.3%) | 1.176 | 0.578-2.390 | 0.719 |
| rs4579322 | Codominant | AA vs TA vs TT | 26(50.0%)/16(30.8%)/10(19.2%) | 24(32.9%)/29(39.7%)/20(27.4%) | - | - | 0.168 |
|  | Allele | A vs T | 68(65.4%)/36(34.6%) | 77(52.7%)/69(47.3%) | 1.693 | 1.008-2.843 | 0.052 |
|  | Dominant | AA+TA vs TT | 42(80.8%)/10(19.2%) | 53(72.6%)/20(27.4%) | 1.585 | 0.670-3.747 | 0.396 |
|  | Recessive | AA vs TA+TT | 26(50.0%)/26(50.0%) | 24(32.9%)/49(67.1%) | 2.042 | 0.983-4.240 | 0.065 |
|  | Heterozygote | TA vs TT | 16(61.5%)/10(38.5%) | 29(59.2%)/20(40.8%) | 1.103 | 0.417-2.923 | 1.000 |
|  | Homozygote | AA vs TT | 26(72.2%)/10(27.8%) | 24(54.5%)/20(45.5%) | 2.167 | 0.846-5.548 | 0.163 |
|  | Additive | AA+TT vs TA | 36(69.2%)/16(30.8%) | 44(60.3%)/29(39.7%) | 1.483 | 0.699-3.148 | 0.348 |

Data are presented as n (%); CI, confidence interval; OR, odds ratio; *p* values were computed using the chi-square test, p＜0.05*;

Codominant model: GG vs AG vs AA; Allele model: G vs A; Dominant model: GG+AG vs AA; Recessive model: GG vs AG + AA;

Heterozygote model: AG vs AA; Homozygote model: GG vs AA. Additive: GG+AA vs AG.
